# Germ Line/Multipotency Genes Show Differential Expression during Embryonic Development of the Annelid *Enchytraeus coronatus*

**DOI:** 10.3390/biology12121508

**Published:** 2023-12-10

**Authors:** Roman P. Kostyuchenko, Daria D. Nikanorova, Artem V. Amosov

**Affiliations:** Department of Embryology, St. Petersburg State University, Universitetskaya nab. 7-9, 199034 St. Petersburg, Russia; daria.nikanorova@mail.ru (D.D.N.); artem221199@mail.ru (A.V.A.)

**Keywords:** *Piwi*, *Vasa*, *Nanos*, germline multipotency program genes, germ line, primordial germ cells, stem cells, posterior growth zone, nervous system, embryonic development, Annelida

## Abstract

**Simple Summary:**

Germline cells are a key cell type in virtually all multicellular animals. They are a source of gametes and are necessary for sexual reproduction. Thus, the establishment and maintenance of the germ line is critical in the life of most animals. Specification of primordial germ cells occurs by involvement of conserved sets of genes. These genes are essential for germline function in a variety of animals, but they are also responsible for pluri- and multipotency and therefore play a broader role in controlling stemness in both germline and somatic tissue.

**Abstract:**

Germ line development and the origin of the primordial germ cells (PGCs) are very variable and may occur across a range of developmental stages and in several developmental contexts. In establishing and maintaining germ line, a conserved set of genes is involved. On the other hand, these genes are expressed in multipotent/pluripotent cells that may give rise to both somatic and germline cells. To begin elucidating mechanisms by which the germ line is specified in *Enchytraeus coronatus* embryos, we identified twenty germline/multipotency genes, homologs of *Vasa*, *PL10*, *Piwi*, *Nanos*, *Myc*, *Pumilio*, *Tudor*, *Boule*, and *Bruno*, using transcriptome analysis and gene cloning, and characterized their expression by whole-mount in situ hybridization. To answer the question of the possible origin of PGCs in this annelid, we carried out an additional description of the early embryogenesis. Our results suggest that PGCs derive from small cells originating at the first two divisions of the mesoteloblasts. PGCs form two cell clusters, undergo limited proliferation, and migrate to the developing gonadal segments. In embryos and juvenile *E. coronatus*, homologs of the germline/multipotency genes are differentially expressed in both germline and somatic tissue including the presumptive germ cell precursors, posterior growth zone, developing foregut, and nervous system.

## 1. Introduction

Germline development that is required for sexual reproduction is very variable and may occur across a range of developmental stages and in several developmental contexts. In many organisms, including model organisms such as *Drosophila*, *Caenorhabditis elegans*, and zebrafish, the precursors of germ cells (primordial germ cells, or PGCs) are segregated from specific cell lineages during cleavage. In others—in mammals, for example—they are specified by inductive interaction between cells at gastrulation stage [1,2]. In a range of animals, including sponges, cnidarians, flatworms, annelids, and ascidians, germ line can even be regenerated anew or re-established in adult animals, from adult stem cells [3,4,5,6,7,8,9,10,11,12,13,14,15,16,17].

A conserved set of mRNAs and proteins is involved in establishing and maintaining germ line. *Piwi*, *Nanos*, *Vasa*, *PL10*, *Pumilio*, *Tudor*, *Boule*, and *Bruno* are among the genes encoding these mRNAs and proteins [2,16]. Initially, *Piwi*, *Nanos*, and *Vasa* homologs, the most conserved genes involved in PGC specification, were thought to be restricted to the germ line. Later, it was shown that they, like other germline markers, can be expressed in multipotent/pluripotent cells that may give rise to both somatic and germline cells [2,6,7,8,9,10,14,15,16,17,18,19,20,21,22,23,24,25,26,27,28].

All these germline/multipotency genes (also called germline multipotency program genes, GMP) [2] are critical for establishment and maintenance of the germ line in the life cycle of most animals and provide a strategy that minimizes the risk of genomic damage associated with replication and mitosis during the cell cycle. Mutations within these genes, or gene knockout, can cause male/female sterility or germ cell/multipotent cell deficiency [22,23,28,29,30,31,32,33,34,35,36,37,38]. GMP genes are mostly involved in the chromatin reorganization, transcriptional repression of somatic cell fate, control of meiosis, and post-transcriptional regulation of various genes during gametogenesis and embryogenesis.

*Vasa* and *Pl10* homologs encode the ATP-dependent RNA DEAD-box helicases, critical components that specify and protect the germline. They regulate translation and amplify piwi-interacting RNAs (piRNAs) [32,39,40,41]. *Piwi* is involved in transposon silencing and RNA interference [2,15,29,32]. *Nanos* is a translational repressor characterized by two CCHC zinc finger motifs [29,30,38]. *Pumilio* is a translational repressor that directly binds to its target mRNAs. Pumilio proteins form multiprotein complexes with other regulatory proteins, such as Boule and Nanos, involved in the maintenance of pluripotent stem cells in various organisms, including humans. Pumilio and Nanos interact in a conserved mechanism for the development and maintenance of germ cells [29,32,35,41,42,43,44,45]. Tudor domain motifs mediate protein–protein interactions required for various DNA-templated biological processes [32,34]. *Bruno* encodes RNA-binding protein that regulates multiple mRNAs involved in gametogenesis and early in embryogenesis [32,33,46,47]. Boule protein exhibits an RNA-recognition motif (RRM) domain and is thus a translational regulator involved in diverse steps of RNA processing, from alternative splicing to mRNA trafficking, stability, decay, and translation [32,36].

Annelids belong to a numerous and widely distributed phylum of segmented worms, with great diversity in morphology and ecology [48]. Although like other spiralians, they display a highly conserved pattern of early development known as “spiral cleavage”, annelid development varies significantly. In contrast to non-clitellate annelids that usually show basic spiral cleavage (homo- or heteroquadrant), gastrulation by epiboly or sometimes by invagination, free-swimming larva, and metamorphosis, clitellates exhibit a wide range of spiral cleavage modifications [49,50,51,52,53]. They also lack the larval stages and metamorphosis. On the other hand, most annelid species are capable of regeneration, sometimes very extensive, including regeneration of gonads, lost body parts, or even the entire body [18,54,55,56]. Asexually reproducing annelids also retain the ability to develop gonads and become sexually mature [8,9,12,24]. All this makes annelids an excellent group for studying germline formation and maintenance, as well as for comparative studies.

Although the study of germline/multipotency genes is of great interest, expression of these markers (mainly *Vasa*, *Piwi*, and *Nanos* homologs) has been investigated in a few annelid species (*Platynereis dumerilii*, *Alitta virens*, *Capitella teleta*, *Tubifex tubifex*, *Enchytraeus japonensis*, *Helobdella robusta*, *Pristina leidyi*, and *Pristina longiseta*). Most of these studies focused specifically on regeneration, several on germline formation or asexual reproduction [6,7,8,9,10,12,18,19,20,24,25,26,27,57,58,59,60,61]. Using a limited set of GMP genes, these studies demonstrated that some combinations of pluripotency genes are expressed in presumptive germ cell precursors and in somatic tissue in embryos, juveniles, or adults.

The species used in this study, *Enchytraeus coronatus*, is a soil clitellate annelid belonging to the family Enchytraeidae. Enchytraeids are the most important organisms in many habitats, dominant both in biomass and abundance [62]. *E. coronatus* is easily maintained in laboratory culture [63,64,65]. Under laboratory conditions, worms reproduce continuously by laying cocoons at any time of year. They are capable of extensive regeneration; however, in contrast to *E. japonensis* incapable of asexual reproduction.

The goal of this work was to study the germline/multipotency gene expression during embryonic development of the annelid *E. coronatus*. Our study represents the first detailed description of the embryonic and juvenile expression of twenty germline/multipotency genes in annelids. We have identified homologs of *Vasa* (*Eco-vasa1*, *Eco-vasa2*), *PL10* (*Eco-pl10-1*, *Eco-pl10-2*), *Piwi* (*Eco-piwi1A*, *Eco-piwi1B*), *Nanos* (*Eco-nanos1*, *Eco-nanos2*), *Myc* (*Eco-myc*), *Pumilio* (*Eco-pumilio2*, *Eco-pumilio3*), *Tudor* (*Eco-tudor1*, *Eco-tudor2*, and *Eco-tudor3*), *Boule* (*Eco-boule1*, *Eco-boule2*), and *Bruno* (*Eco-bruno1*, *Eco-bruno2*, *Eco-bruno3*, and *Eco-bruno4*), and characterized their expression using whole-mount in situ hybridization. To answer the question of the possible origin of PGCs in this annelid, we carried out an additional description of the early embryogenesis. During embryonic development and in juvenile *E. coronatus*, homologs of the germline/multipotency genes are differentially expressed in both germline and somatic tissue including the presumptive germ cell precursors, posterior growth zone, developing foregut, and nervous system.

## 2. Materials and Methods

### 2.1. Animal Material and Fixation

The laboratory culture of the oligochaete *Enchytraeus coronatus* was obtained previously from Dr. Achim Paululat. Worms were cultured in Petri dishes on 2% Agar in 0.05% Knopp solution (4.2 мM Ca(NO_3_)_2_*4 H_2_O, 1 мM MgSO_4_*7 H_2_O, 1.8 мM KH_2_PO_4_, 2.5 мM KNO_3_, 1.6 мM KCl, and spurs of FeCl3*6 H_2_O) [64] at 18 °C. Animals were fed once a week with rolled oats and transferred to new agar plates every 3 weeks as previously described [64]. Under these conditions, worms reproduce continuously by laying cocoons, which contain one to several eggs each, at any time of year.

Cocoons were collected from fresh agar plates. For DIC analysis of unlabeled embryos, cocoons were fixed in 4% PFA in PTw (PBS/0.1% Tween-20) overnight at 4 °C. After washing in PBS, specimens were transferred through a graded glycerol series (25%, 50%, and 75% glycerol in PBS) and embedded in glycerol/PBS solution (9:1).

To obtain materials for in situ hybridization and immunohistochemistry, cocoons were put in 4% PFA in PTw and irradiated using a conventional microwave oven for 10 sec. To prevent overheating, the 2.0 mL Eppendorf safe-lock tubes containing the specimens were surrounded with ice during exposure to microwaves. The material was then fixed in a fresh portion of 4% PFA in PTw for 1 h at room temperature and washed in PTw. The cocoon shell was removed manually using dissecting needles and a micro scalpel under a Leica EZ4 stereo microscope. After cocoon removing, embryos were postfixed in 4% PFA in PTw overnight at +4 °C and stored in MeOH at −20 °C. Juvenile worms were collected from fresh culture dishes, fixed in 4% PFA in PTw overnight at +4 °C, and stored in MeOH at −20 °C.

### 2.2. Immunohistochemistry

To describe in more detail the development of *E. coronatus*, immunohistochemical studies were carried out according to the previously published protocol [66]. After storage in MeOH, fixed embryos were hydrated to PTw and permeabilized with 0.1% Triton-X in PBS (PBT). Specimens were then preincubated in 5% normal sheep serum (Sigma, Cat. #S2263, Burlington, MA, USA) in PBT for 1 h at room temperature, and incubated overnight at 4 °C in PBT with mouse anti-β-tubulin monoclonal antibody (Sigma, Cat. #T4026) diluted 1:200 in 2.5% normal sheep serum. Embryos were then washed in PBT several times and incubated in PBT with Cy5-conjugated anti-mouse antibody (diluted 1:400; Jackson ImmunoResearch, West Grove, PA 19390, United States, Cat. #715-175-150) and DAPI (1 mkg/mL) for 2 h at room temperature. After washing with PTw, specimens were embedded in 90% glycerol in PBS and examined by confocal laser scanning microscopy.

### 2.3. Sequence Retrieval, Gene Cloning, and Phylogenetic Analysis

To study the expression of the germline/multipotency genes, we cloned their orthologs from the annelid *E. coronatus*, which is easily maintained in laboratory culture. The sequences of *Vasa*, *Pl10*, *Piwi*, *Nanos*, *Myc*, *Pumilio*, *Tudor*, *Boule*, and *Bruno* homologs were retrieved from an unannotated *E. coronatus* transcriptome database (local resource; the transcriptome was deciphered using Illumina HiSeq 2500 sequencing). Fragments for *Eco-vasa1*, *Eco-vasa2*, *Eco-pl10-1*, *Eco-pl10-2*, *Eco-piwi1A*, *Eco-piwi1B*, *Eco-nanos1*, *Eco-nanos2*, *Eco-myc*, *Eco-pumilio2*, *Eco-pumilio3*, *Eco-tudor1*, *Eco-tudor2*, *Eco-tudor3*, *Eco-boule1*, *Eco-boule2*, *Eco-bruno1*, *Eco-bruno2*, *Eco-bruno3*, and *Eco-bruno4* genes were amplified by PCR with gene-specific primers and mixed-stage embryo cDNA prepared with a SMARTer RACE cDNA amplification kit (Clontech, Cat. #634923, Mountain View, CA, USA). All primers are given in the Supplementary Materials (Table S1). The amplified gene fragments were cloned into pCRII vectors (Invitrogen, Cat. #K4600-01, Invitrogen, MA, USA) that were then used in the transformation of chemically competent *E. coli* (One Shot^®^ TOP10; Invitrogen, Cat. #K4600-01). Plasmids with correct inserts were checked by sequencing. The identity of cloned gene fragments was confirmed through phylogenetic analysis (see below). As a result, except for *Eco-vasa1*, *Eco-pl10-1*, *Eco-pl10-2*, *Eco-tudor1*, *Eco-tudor2*, and *Eco-tudor3*, all amplified fragments include complete CDS, and 5′ and 3′ UTR. The sequences of *Eco-vasa1*, *Eco-vasa2*, *Eco-pl10-1*, *Eco-pl10-2*, *Eco-piwi1A*, *Eco-piwi1B*, *Eco-nanos1*, *Eco-nanos2*, *Eco-myc*, *Eco-pumilio2*, *Eco-pumilio3*, *Eco-tudor1*, *Eco-tudor2*, *Eco-tudor3*, *Eco-boule1*, *Eco-boule2*, *Eco-bruno1*, *Eco-bruno2*, *Eco-bruno3*, and *Eco-bruno4* genes were deposited in GenBank with the accession numbers OR750672–OR750691. The obtained plasmids were used for RNA probe synthesis.

For phylogenetic analysis, the homologous proteins of interest were searched in the NCBI protein database. To establish homology, we employed BLASTp. Muscle algorithm [67] integrated into Unipro UGENE v47.0 software [68] was used to perform multiple sequence alignment. The following specific strategy for phylogenetic analysis varied depending on the gene under investigation: Conservative domains identified by PROSITE (https://prosite.expasy.org/, accessed on 25 December 2021) were used in the phylogenetic analysis of *Piwi, Boule, Pumilio, Nanos, Myc*, and *Bruno* homologs. Gblocks-curated alignments were used for *Vasa* and *Pl10* homologs. The full-length alignment was manually curated and used for the phylogenetic analysis of *Tudor* homologs.

To infer the evolutionary relationships among the sequences, we performed Bayesian phylogenetic analysis using MrBayes 3.2.7 (https://www.phylo.org/, accessed on 8 October 2023) [69]. The LG model was chosen and rate variation across sites was fixed to “invgamma”. Four Markov Chain Monte Carlo (MCMC) chains were run for 400,000 generations, sampling every 500 generations, with a burn-in value of 0.25. Finally, a 50% majority rule consensus tree was constructed. R version 4.3.1, in conjunction with RStudio version 2023.06.0 Build 421 was used for visualization (Packages: *ggplot2*, *ggtree*, and *treeio*). To improve the clarity and interpretability of the phylogenetic trees, we applied a branch-length rescaling technique. This rescaling focused on the branch lengths of the outgroups in the trees for specific genes, namely *Vasa*, *Piwi*, *Myc*, *Pumilio*, *Bruno*, *Boule*, and *Tudor* homologs. The rescaling was accomplished by adjusting the branch lengths relative to the mean branch length in each respective tree (Supplementary Materials, Figures S1–S8).

### 2.4. Whole-Mount in Situ Hybridization

Whole-mount in situ hybridization (WMISH) was carried out as previously described [66,70] except that fixations (see 2.1. Animal material and fixation) and the step for Proteinase K digestion were modified. Specimens were rehydrated from MeOH, rinsed several times in PTw, treated with Proteinase K (100µg/mL; Merck, Cat. #1.24568.0100) for 0.5–1 min at +22 °C, rinsed twice in glycine (2mg/mL), and postfixed with 4% PFA in PTw for 20 min. Prior to the pre-hybridization step, samples were washed several times in PTw. The templates for the *Eco-vasa1*, *Eco-vasa2*, *Eco-pl10-1*, *Eco-pl10-2*, *Eco-piwi1A*, *Eco-piwi1B*, *Eco-nanos1*, *Eco-nanos2*, *Eco-myc*, *Eco-pumilio2*, *Eco-pumilio3*, *Eco-tudor1*, *Eco-tudor2*, *Eco-tudor3*, *Eco-boule1*, *Eco-boule2*, *Eco-bruno1*, *Eco-bruno2*, *Eco-bruno3*, and *Eco-bruno4* digoxigenin-labeled RNA probes (antisense and sense) were, respectively, ~1336 bp (positions 141–1477 in GenBank OR750672), ~1559 bp (positions 1274–2833 in GenBank OR750673), ~1464 bp (positions 21–1485 in GenBank OR750674), ~1419 bp (positions 957–2376 in GenBank OR750675), ~1428 bp (positions 1371–2799 in GenBank OR750676), ~1939 bp (positions 543–2482 in GenBank OR750677), ~810 bp (positions 343–1153 in GenBank OR750678), ~1107 bp (positions 82–1189 in GenBank OR750679), ~1261 bp (positions 95–1356 in GenBank OR750680), ~1490 bp (positions 1485–2975 in GenBank OR750681), ~1412 bp (positions 312–1724 in GenBank OR750682), ~1438 bp (positions 1789–3227 in GenBank OR750683), ~1392 bp (positions 6105–7497 in GenBank OR750684), ~1347 bp (positions 249–1596 in GenBank OR750685), ~1156 bp (positions 126–1282 in GenBank OR750686), ~771 bp (positions 578–1349 in GenBank OR750687), ~882 bp (positions 125–1007 in GenBank OR750688), ~873 bp (positions 60–933 in GenBank OR750689), ~1171 bp (positions 127–1298 in GenBank OR750690), and ~1020 bp (positions 71–1091 in GenBank OR750691). After hybridization with antisense digoxigenin-labeled RNA probes and washing steps, specimens were incubated with anti-digoxigenin AP antibodies (1:2500; Roche, Cat. #1093274910), washed, and stained with NBT/BCIP (Roche, Cat. #11383213001/11383221001). Specimens were then washed again and mounted in 90% glycerol. In situ hybridization with the sense DIG-labeled riboprobes was used as a negative control.

### 2.5. Data Visualization

After in situ hybridization and fixation for DIC analysis, imaging of the mounted glycerol specimens was conducted using DIC optics on an Axio Imager D1 microscope (Carl Zeiss, Oberkochen, Germany). Pictures were taken with an AxioCam ICc3 digital camera using the AxioVision 4.8 software (Carl Zeiss, Oberkochen, Germany). After immunohistochemistry, specimens were imaged using a Leica SP5X confocal laser scanning microscope (Leica, Wetzlar, Germany). Z-stacks with 1.0 mkm steps were acquired using the Leica LAS X Office software. The artworks were created in MS PowerPoint (Microsoft Office 2013) and Adobe Photoshop CS5.

## 3. Results

### 3.1. Life Cycle of the Annelid Enchytraeus Coronatus

**Figure 1:**
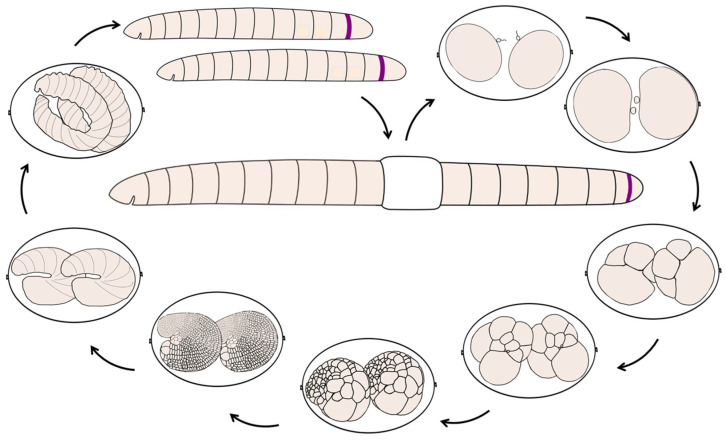
Scheme of the life cycle and development of the annelid *Enchytraeus coronatus*. *E. coronatus* are simultaneous hermaphrodites that undergo sexual reproduction, usually with cross-fertilization. Mature animals have gonads located in segments X (testes) and XI (ovaries). A clitellum, formed by epidermal cells of segments XI–XIII, secrete a lemon-shaped cocoon that provides a microenvironment for embryonic development. After the deposition of oocytes and sperm into a cocoon, fertilization occurs, and oocytes undergo a second meiotic division. There are no larval stages, and embryos develop directly into juveniles over anywhere from one week to two weeks, depending on the environmental conditions (approximately 9 days at 18 °C). Embryos exhibit a modified version of unequal spiral cleavage. As a result, the embryo forms ectoderm- and mesoderm-specific large cells called teloblasts, which produce a germinal band. Gastrulation occurs by epiboly and convergent extension. During development, embryos undergo dramatic changes in shape and become elongated. At the end of gastrulation, segments appear from anterior to posterior. Later, the elongation of an individual occurs by sequential addition of segments from a posterior growth zone. Hatched juveniles are similar to adults, but do not have clitellum or developed gonads. They feed, grow, and become sexually mature in approximately three weeks. The scheme is not to scale.

*E. coronatus* are soil clittelate annelids, belonging to the Oligochaete. This species is easy to handle and has relatively short generation times and high reproductive rates [63]. Worms are white in color and have transparent cuticle. Adult animals are from eight to twelve mm in length and exhibit about 28–36 segments. They can be easily recognized through the presence of a clitellum, formed by epidermal cells of segments XI–XIII. The animals reproduce sexually, laying transparent cocoons with the eggs (Figure 1). The number of eggs/embryos within one cocoon can vary and ranges from 1 to 7. *E. coronatus* are simultaneous hermaphrodites, capable of self-fertilization [64], however, they usually exhibit cross-fertilization. The gonads are located in segments X (testes) and XI (ovaries). The gland cells of the clitellum secrete the cocoon shell. Fertilization of the eggs occurs within the cocoon and leads to oocyte maturation, zygote formation, and the further development of the embryos. At 18 °C, embryonic development until hatching from the cocoon lasts approximately 9 days. Oligochaete *E. coronatus* is a direct developer, that is, development occurs without a larval stage. Hatched juvenile worms are similar to adults, but do not have clitellum or developed gonads. They feed and grow, increasing the number of segments due to the growth zone and becoming sexually mature in approximately three weeks.

### 3.2. Development of the Annelid E. coronatus

The embryonic development of *E. coronatus* has been previously described using scanning electron microscopy and histology [64]. Here we provide a summary of the development and an additional description of the early embryogenesis. Developmental stages are designated according to [64].

During the first day after cocoon deposition (0–24 h, Stage 1), early cleavage and the formation of mesoteloblasts and proteloblasts are observed.

**Figure 2:**
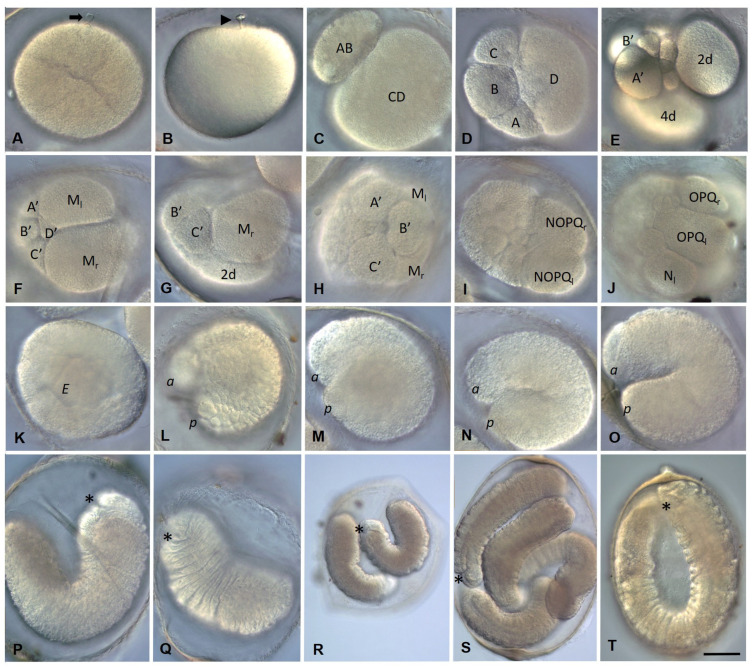
Development of the annelid *E. coronatus*. DIC of the embryos inside the cocoons. (**A**–**I**) Zygote formation and blastomere cleavage during stage 1. (**A**) Fertilization cone formation (arrow) during sperm penetration into the oocyte. (**B**) Release of the second polar body (arrowhead). (**C**) Two-cell embryo, view from the animal pole. (**D**) Four-cell embryo, view from the animal pole. The D blastomere is the largest cell of the embryo. (**E**) The primary somatoblast (2d) and secondary somatoblast (4d), lateral view. (**F**–**H**) Mesodermal teloblasts (daughter cells of the secondary somatoblast), view from the posterior vegetal (**F**), lateral view, dorsal down in (**G**), anterior animal pole in (**H**). (**I**) Bilateral pair of NOPQ proteloblasts (products of the primary somatoblast), dorsal view. (**J**) At the end of stage 2, the embryo becomes round-shaped. (**K**,**L**) Stage 3 is characterized by elongation of the germband. All teloblasts retain their position at the posterior end. (**M**–**O**) During stage 4, embryos elongate and become U-shaped. (**P**) At stage 5, embryos start moving within the cocoon and segments can be seen at the anterior part of the embryo. (**Q**,**R**) Additional segments form progressively from anterior to posterior during stage 6. (**S**,**T**) During days 7–9, the worms become fully developed and ready to hatch. *E* marks the endoderm; *a* and *p* mark the anterior and posterior ends, respectively; the asterisk marks the mouth position. Scale bar, 50 µm for all panels except (**R**). Scale bar in (**R**), 85 µm.

After the eggs and sperm are deposited into a cocoon, fertilization occurs. During sperm penetration into the oocyte, a fertilization cone is formed (Figure 2A). Sperm entry triggers the oocyte to complete the second meiotic division. As a result, a second polar body is released and a zygote is formed (Figure 2B). Zygotes are yolk-rich. They vary in shape and size but usually have an oval, slightly irregular shape and a diameter of about 150–160 µm. During egg maturation and zygote formation, clear morphological signs of ooplasmic segregation, such as the cytoplasmic movement or accumulation of the pole plasm (teloplasm), are not observed. However, the egg changes shape several times during the second meiotic division.

**Figure 3:**
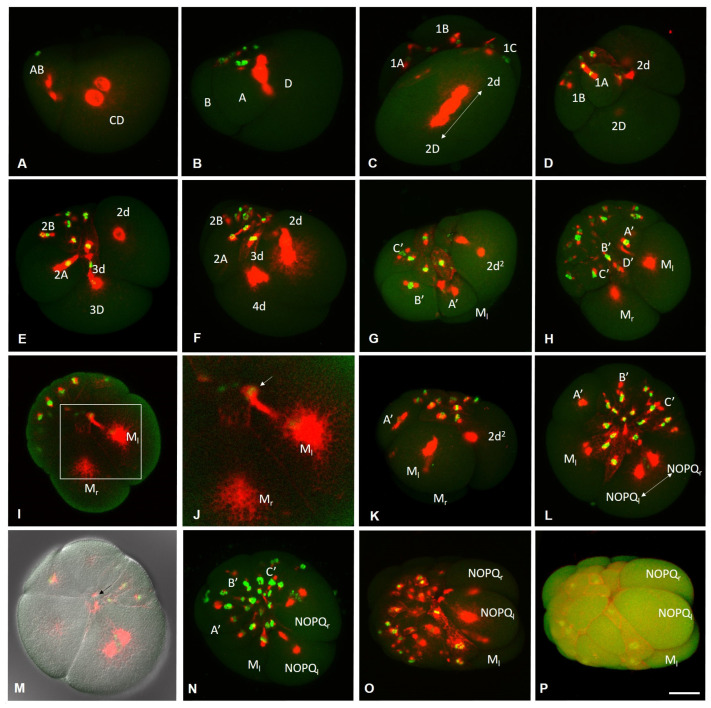
Development of the annelid *E. coronatus* during stage 1. Immunohistochemistry against β-tubulin (red) and DNA staining with DAPI (green). Laser scanning confocal microscopy. Maximum (**A**–**O**) and transparent projections (**P**) of confocal Z-stacks. (**A**) Two-cell embryo, lateral view. The CD and AB blastomeres start to divide into a smaller C and a larger D blastomere, and into the same-sized A and B blastomeres, respectively. (**B**) Transition to the 9-cell embryo, lateral view. The D blastomere generates a small micromere 1d and a large macromere 1D. (**C**) The 1D blastomere divides into the slightly larger 2D macromere and the 2d micromere, dorsal view. (**D**) 2d is born prior to other second-quartet micromeres, lateral view. (**E**) The 2D macromere gives rise to the 3D macromere and 3d micromere by asymmetric division, lateral view. (**F**) The secondary somatoblast (4d) starts to produce the mesoteloblasts, lateral view. Simultaneously, the primary somatoblast 2d undergoes a highly unequal division, giving off a smaller daughter cell toward the anterior side of the larger daughter cell. (**G**–**K**) Mesodermal teloblasts, animal-lateral view (**G**), ventral view, different focal plane (**H**–**J**), and lateral view in (**K**). (**H**) Mesoteloblasts undergo highly unequal division. (**I**,**J**) The same embryo as in (**H**), deep focal plane. (**J**) An enlarged view of the boxed region shown in (**I**) highlighting a small daughter cell with a high nucleo-cytoplasmic ratio (arrow). (**L**) Formation of the NOPQ proteloblasts, view from animal pole. (**M**) The same embryo as in (**L**), deep focal plane. Arrow marks contacts between blastomeres in the stereoblastula. (**N**–**P**) The NOPQ proteloblasts divide twice by highly unequal divisions cutting off the smaller cells anteriorly. Scale bar, 50 µm for all panels except (**I**). Scale bar in (**I**), 20 µm.

Embryos of *E. coronatus* undergo a modified version of spiral cleavage (Figure 2 and Figure 3). Early cleavage is characterized by unequal and slightly asynchronous divisions. During the earliest steps of development, cell cycle length varies between 1 h 20 min and 1 h 40 min at 18 °C. The zygote undergoes an extremely unequal cleavage and gives rise to a small AB- and a large CD-cell (Figure 2C and Figure 3A). The CD blastomere divides into a smaller C and a larger D blastomere. In approximately 20 min, AB divides equally into A and B blastomeres. At the four-cell stage, the D blastomere is the largest cell of the embryo (Figure 2D). Next, it divides again, producing a small micromere 1d and a large macromere 1D (Figure 3B). After the appearance of the first-quartet micromeres in the quadrants A, B, and C, the 1D blastomere divides into the slightly larger 2D macromere and the 2d micromere (Figure 3C,D). Thus, 2d is born prior to other second-quartet micromeres. It is a precursor of the body’s ectoderm and called the primary somatoblast.

During subsequent cell divisions of the embryo, the number of micromeres increases. According to the genealogy of blastomeres accepted for clitellates, we will designate the macromeres in quadrants A, B, and C as A’, B’, and C’. The 2D macromere divides twice (Figure 2E and Figure 3E,F). The second division is extremely unequal and produces a very large 4d micromere, the second somatoblast, and a very small 4D macromere (D’). The latter is located ventrally, between macromeres A’, B’, C’, and micromere 4d (Figure 2F and Figure 3H). The primary somatoblast 2d undergoes a highly unequal division, giving off a smaller daughter cell toward the anterior side of the larger daughter cell (Figure 3F,G). Simultaneously, the 4d cell further divides bilaterally, into the two mesoteloblasts (M-cells), the largest cells of the embryo, which are located ventrally on both sides of the embryo (Figure 2F,G and Figure 3G,H,K). The M-cells undergo two rounds of highly unequal divisions, giving off smaller daughter cells anteriorly (Figure 3I–K). These small cells exhibit a high nucleo-cytoplasmic ratio. 2d^2^, the larger daughter cell that results from the division of the 2d blastomere, then divides equally into a bilateral pair of NOPQ proteloblasts (Figure 2I and Figure 3L–P). The proteloblasts generate the majority of the body and mark the dorsal posterior pole of the embryo. The blastula is a stereoblastula and has no blastocoel (Figure 3M). The descendants of macromeres represent the prospective endoderm.

**Figure 4:**
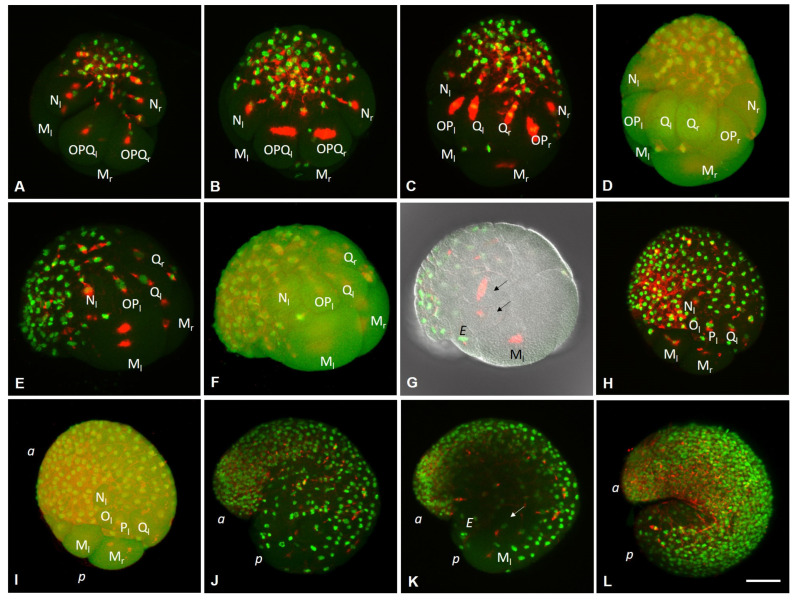
Development of the annelid *E. coronatus* during stages 2–4. Immunohistochemistry against β-tubulin (red) and DNA staining with DAPI (green). Laser scanning confocal microscopy. Maximum (**A**–**C**,**E**,**G**,**H**,**J**–**L**) and transparent projections (**D**,**F**,**I**) of confocal Z-stacks. (**A**) Bilateral pairs of N ectoteloblasts and OPQ proteloblasts are located ventrolaterally and dorsally, respectively. They undergo highly unequal divisions and generate rows of n- and opq-blast cells. (**B**) The OPQ cells divide almost equally into the dorsally located ectoteloblasts Q and lateral proteloblasts OP. (**C**–**F**) Ectoteloblasts and proteloblasts generate bandlets of blast cells by asymmetric teloblastic divisions, dorsal (**C**,**D**) and dorsolateral view (**E**,**F**). (**G**) Divisions of the large m-blast cells (arrows) at the left side of the embryo, the same embryo as in (**E**,**F**), deep focal plane. (**H**–**L**) Elongation of the germbad during gastrulation and organogenesis, dorsolateral (**H**–**K**) and ventrolateral (**L**) view. (**J**,**K**) The same embryo, deep focal plane in (**K**). (**L**) The posterior part of the embryo is less differentiated and the posteriorly located teloblasts continue to generate undifferentiated cells by asymmetric divisions. *E* marks the endoderm; *a* and *p* mark the anterior and posterior ends of the embryo, respectively. Scale bar, 50 µm for all panels.

On the second day after cocoon deposition (24–48 h, stage 2), the formation of the germband occurs. On either side of the embryo, the NOPQ proteloblasts divide synchronously into the four ectoteloblasts. During several rounds of cell division, ectoteloblasts N, Q, and, finally, O and P are produced sequentially. After their appearance, both NOPQ divide twice by highly unequal divisions cutting off the smaller cells anteriorly (Figure 3N,O). Bilateral pairs of N ectoteloblasts and OPQ proteloblasts, which are then generated, are located ventrolaterally and dorsally, respectively (Figure 2J and Figure 4A). Each N teloblast gives rise to a row of n-blast cells by asymmetric teloblastic divisions (n-bandlet) (Figure 4A,B). Simultaneously, the OPQ cells undergo highly unequal divisions, twice cutting off smaller cells from themselves, first anteriorly and then posteriorly. Next, each OPQ cell divides almost equally into the dorsally located ectoteloblast Q and lateral proteloblast OP (Figure 4B–D). The Q ectoteloblasts produce q-blast cells via asymmetric teloblastic divisions. The OP cells then generate several smaller cells anteriorly and finally cleave equally into ectoteloblasts O and P, giving rise to the o- and p-bandlets (Figure 4C–F,H,I). The two q-bandlets are separated at the dorsal midline by a single row of cells generated by divisions of 2d^1^ and its derivatives.

Mesoteloblasts give rise to two rows of m-blast cells. Although they continue to divide teloblastically, the divisions are less unequal, and the resulting m-blast cells are much larger than the products of the first two divisions (Figure 4G). The bandlets of mesodermal and ectodermal teloblast descendants form the germband at the dorsal side of the embryo. At the end of the second day of development, the embryo becomes round-shaped and exhibits distinct anterior and posterior poles, formed by the descendants of the micromeres and teloblasts.

The third day after cocoon deposition (48–72 h, stage 3) is characterized by elongation of the germband. All teloblasts continue to generate blast cells by asymmetric divisions; however, they retain their position at the posterior pole. The germband begins to curve convexly around the ventral endodermal cells (Figure 2L and Figure 4H,I). Ectoteloblasts divide synchronously at this stage (Figure 4H,I). During germband elongation, the M-cells and ventral endodermal cells are overgrown by the ectoteloblasts and dividing blast cells (Figure 2L). The lateral borders of ectodermal cells are characterized by high cell density (Figure 4H,I). Thus, gastrulation starts with epiboly. At this stage, the stomodeum begins to form at the anterior ventral side of the embryo.

During the fourth day after cocoon deposition (72–96 h, stage 4), dynamic changes in embryonic body morphology as well as in organogenesis are observed. Starting at the anterior pole of the embryo, dorsolateral ectodermal cells migrate from both sides of the embryo, overgrowing the endodermal cells from the dorsal to the ventral side. The leading marginal cells meet each other at the ventral midline, where they form the ventral nerve cord from anterior to posterior. Embryos elongate and become U-shaped (Figure 2M–O and Figure 4J–L). After some grade of convergent extension, gastrulation is completed at the anterior end. Simultaneously, the stomodeal plate invaginates and the mouth finally forms. According to [64,65], the cerebral ganglion forms at the beginning of this stage. It is located dorsally, just anterior to the mouth opening. The cerebral ganglion and the developing ventral nerve cord become connected to each other by esophageal connectives. Although the rapid progress of development is obvious at the anterior end, the posterior part of the embryo is less differentiated. Posteriorly located teloblasts continue to generate undifferentiated cells by asymmetric divisions.

On the next day (96–120 h, stage 5), the embryos start moving within the cocoon. The elongated body of the embryo loses its ventrally curved shape. The posterior part of the embryo is still less differentiated. Although the progress of overgrowing the endoderm by the ecto- and mesoderm is obvious, the leading marginal ectodermal cells do not yet meet each other at the ventral midline at the posterior pole. The ectoteloblasts distinguishable at the beginning of this day can no longer be identified at the end of this stage. Eight segments appear at the anterior part of the embryo (Figure 2P).

The next day (120–144 h, stage 6) is characterized by the end of overgrowing the endoderm by the ecto- and mesoderm. The ectodermal cells of either side meet each other at the ventral midline of the posterior part of the embryo. Additional segments form progressively from anterior to posterior (Figure 2Q,R).

During days 7–9, the animals become fully developed. They consist of 15–16 segments and form seta immediately before hatching (Figure 2S,T).

### 3.3. Expression of Germ Line/Multipotency Genes during Embryonic Development and at Juvenile Stage of *E. coronatus*

In this study, we identified homologs of *Vasa* (*Eco-vasa1*, *Eco-vasa2*), *PL10* (*Eco-pl10-1*, *Eco-pl10-2*), *Piwi* (*Eco-piwi1A*, *Eco-piwi1B*), *Nanos* (*Eco-nanos1*, *Eco-nanos2*), *Myc* (*Eco-myc*), *Pumilio* (*Eco-pumilio2*, *Eco-pumilio3*), *Tudor* (*Eco-tudor1*, *Eco-tudor2*, and *Eco-tudor3*), *Boule* (*Eco-boule1*, *Eco-boule2*), and *Bruno* (*Eco-bruno1*, *Eco-bruno2*, *Eco-bruno3*, and *Eco-bruno4*) from the annelid *E. coronatus*. Expression of these germline/multipotency genes was examined throughout embryonic development and at the juvenile stage of E. coronatus by whole-mount in situ hybridization (WMISH).

#### 3.3.1. *Eco-vasa1* and *Eco-vasa2* Embryonic and Juvenile Expression Patterns

**Figure 5:**
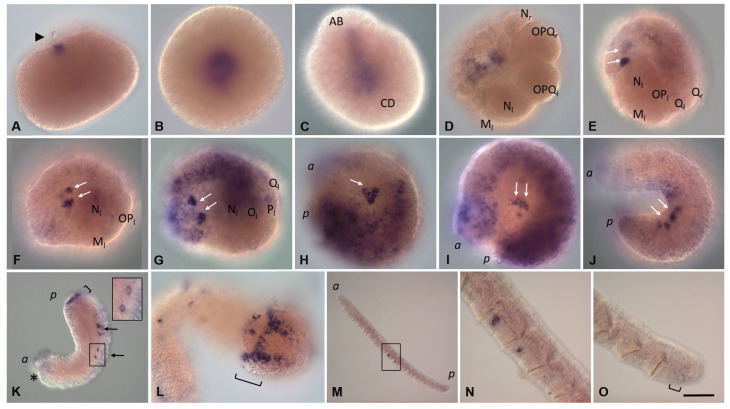
*Eco-vasa1* expression patterns during stage 1 (**A**–**D**), stage 2 (**E**–**G**), stage 3 (**H**), stage 4 (**I**,**J**), stage 5 (**K**), stage 7 (**L**), and in juvenile worms (**M**–**O**). Lateral view, except (**C**) (view from animal pole), (**D**) (dorsal view), and (**M**) (view from the caudal end). (**A**) Transcripts in the yolk-free ooplasm during the second meiotic division and (**B**) zygote. (**C**) Two-cell embryo. (**D**) At the end of stage 1, transcripts are detected at low levels in the ectoteloblast lineage (N, OPQ) and animal micromeres. (**E**–**G**) Robust expression in the germband cells and two small bilateral groups of deep cells, putative PGCs. (**E**) Expression in teloblasts. (**H**,**I**) The number of *Eco-vasa1*-positive cells in the clusters increases from two to six in each. (**J**) Expression gradually weakens in the germband cells and the descendants of micromeres. (**J**,**K**) PGCs seem likely migrating. (**K**,**L**) Expression in the developing posterior growth zone. (**M**) Expression in germline cells in the gonadal segments. (**N**) Enlarged view of the boxed region shown in (**M**). (**O**) Expression in the developing posterior growth zone. The arrowhead marks the second polar body; the arrows mark putative PGCs; the asterisk marks the mouth position; the square bracket marks the posterior growth zone; the *a* and *p* mark the anterior and posterior ends, respectively. Scale bar, 50 µm for all panels except (**K**,**M**). Scale bar in (**K**), 90 µm, in (**M**), 170 µm.

*Eco-vasa1* transcripts are detected in the yolk-free cytoplasm of an oocyte. After the completion of the second meiotic division, they move from the animal pole to the center of the egg (Figure 5A,B), accumulating in the perinuclear cytoplasm. During the early stages of cleavage, transcripts are first detected in the AB and CD blastomeres (Figure 5C) and then in all four founder cells of the embryonic quadrants. At the four-cell embryo stage, the largest blastomere (D) inherits most of the yolk-free cytoplasm and *Eco-vasa1 mRNA* (not shown). Later, expression disappears in most cells, but *Eco-vasa1* transcripts are found at very low levels in the ectoteloblast lineage and animal micromeres. Robust expression of this gene is detected in the ectodermal blasts cells forming the germband (Figure 5D,F,G). M mesoteloblasts show no evidence of *Eco-vasa1* expression, but a clear transcript signal was detected in two small bilateral groups of deep cells at stage 2. These small cells are located anterior to the mesoteloblasts and are characterized by a high nucleo-cytoplasmic ratio (Figure 5E–G). Taking into account all these features and subsequent migration to the gonadal segments, it can be assumed that these are the primordial germ cells (PGCs). As the germband elongates, two clusters of these deep *Eco-vasa1*-positive cells begin to move posteriorly to the ventral side. The number of cells in the clusters increases from two to six in each (Figure 5H,I). At this stage, transcripts are also found in the mesoteloblasts (Figure 5I). Simultaneously, the level of expression gradually weakens in the germband cells and the descendants of micromeres, which participate in the formation of head structures. Clusters of PGCs disappear, and the cells that were previously part of them become localized separately on the ventral side of the middle part of the embryo (Figure 5J,K). These cells seem likely migrating. When gastrulation is complete, expression in the ectoderm and mesoderm gradually disappears, except for the most caudal region of the embryo (Figure 5J–L). At stage 5, a weak expression of *Eco-vasa1* can be detected in the embryonic pharynx (Figure 5K). At the end of embryonic development, expression is shown in the cells of the posterior growth zone and the PGCs (Figure 5L). In juveniles, *Eco-vasa1* transcripts mark the germ cells in the gonadal segments 10 and 11 (Figure 5M,N). In the posterior growth zone, expression is detected at a low level in a few cells (Figure 5O).

**Figure 6:**
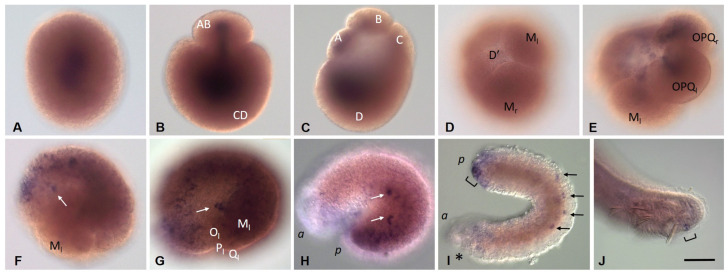
*Eco-vasa2* expression patterns during stage 1 (**A**–**E**), stage 2 (**F**), stage 3 (**G**), stage 4 (**H**), stage 5 (**I**), and in juvenile worms (**J**). Lateral view, except (**B**,**C**) (view from animal pole), **D** (ventral view), and **E** (dorsolateral view). (**A**) Zygote. (**B**) Two-cell embryo. (**C**) Four-cell embryo. (**D**) Transcripts are not shown in mesoteloblasts. (**E**) Expression in the proteloblasts and ectoteloblasts. (**F**,**G**) Expression in the germband cells, descendants of the animal micromeres, and two small bilateral groups of deep cells, putative PGCs. (**H**) Expression gradually weakens in germband cells and descendants of micromeres. (**H**,**I**) PGCs seem to be migrating. (**I**) Expression in the developing posterior growth zone. (**J**) Expression in the posterior growth zone. Arrows mark putative PGCs; an asterisk marks the mouth position; a square bracket marks the posterior growth zone; *a* and *p* mark the anterior and posterior ends, respectively. Scale bar, 50 µm for all panels except (**I**). Scale bar in (**I**), 75 µm.

*Eco-vasa2* transcripts are also found in the yolk-free cytoplasm of an oocyte and an uncleaved zygote (Figure 6A). They are segregated into both blastomeres at the two-cell embryo stage (Figure 6B). Most of the *Eco-vasa2 mRNA* then occurs in the D blastomere (Figure 6C). Later, expression is detected in the proteloblasts and ectoteloblasts, while the M-cells seem likely to be free of *Eco-vasa2* mRNA (Figure 6D). Later, *Eco-vasa2* expression is observed in a more discrete pattern with three distinct domains, including cells of the germband, descendants of the animal micromeres, and small deep cells located anterior to the mesoteloblasts on either side of the embryo (Figure 6F). The time of appearance of these cells and their position are the same as for putative PGCs expressing *Eco-vasa1*. These deep *Eco-vasa2*-positive cells move first posteriorly to the ventral side in two clusters and then migrate (Figure 6G–I). At the end of embryonic development, *Eco-vasa2* expression in these cells, as well as in other mesodermal and ectodermal cells, except for the developing posterior growth zone, disappears. In juveniles, *Eco-vasa2* transcripts are found at low levels in cells of the posterior growth zone (Figure 6J).

#### 3.3.2. *Eco-pl10-1* and *Eco-pl10-2* Embryonic and Juvenile Expression Patterns

**Figure 7:**
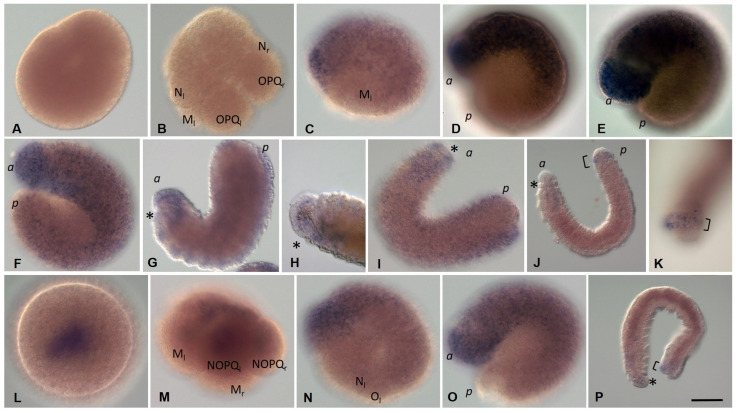
*Eco-pl10-1* (**A**–**K**) and *Eco-pl10-2* (**L**–**P**) expression patterns during stage 1 (**A**,**B** and **L**,**M**), stage 2 (**C**,**N**), stage 3 (**D**,**E**,**N**), stage 4 (**E**,**O**), stage 5 (**G**,**H**), stage 6 (**I**), stage 8 (**J**,**P**), and in juvenile worms (**K**). Lateral view, except (**B**) and (**M**) (dorsal and dorsolateral, respectively), and (**I**) (ventral view). (**A**,**L**) Zygote. (**B**) No *Eco-pl10-1* expression is detected at the OPQ proteloblast stage. (**C**,**N**) Expression in the germband cells and descendants of the animal micromeres. (**D**,**E**) Robust *Eco-pl10-1* expression within the anterior part of the embryo, while transcripts of this gene are detected at low levels in the posterior region of the germband. (**F**,**G**,**O**) Expression of both *Pl10* orthologs gradually weakens in germband cells and descendants of micromeres. (**G**–**I**) *Eco-pl10-1* expression in the developing foregut. (**J**,**K**,**P**) Expression in the developing posterior growth zone. (**M**) *Eco-pl10-2* transcripts are detected in all teloblast lineages (mesodermal and ectodermal). The asterisk marks the mouth position; the square bracket marks the posterior growth zone; *a* and *p* mark the anterior and posterior ends, respectively. Scale bar, 50 µm for all panels except (**H**,**J**), and (**P**). Scale bar in (**H**), 40 µm; in (**J**) and (**P**), 90 µm.

At stage 1, no expression of *Eco-pl10-1* gene is detected in oocytes or any blastomeres (Figure 7A,B). At stage 2, transcripts appear in the descendants of the animal micromeres and the germband cells (Figure 7C). Expression becomes robust in these cells within the anterior part of the embryo during the next stage, while low levels of diffuse expression are detected in the posterior region of the germband (Figure 7D,E). One day later, the levels of *Eco-pl10-1* transcripts decrease in anteroposterior progression (Figure 7F). At stages 5–8, *Eco-pl10-1 mRNA* is observed in the developing foregut, brain, and at the most posterior region of the embryo (Figure 7G–J). In juveniles, weak expression is detected in the posterior growth zone (Figure 7K).

*Eco-pl10-2* transcripts are found in the yolk-free cytoplasm of an oocyte and an uncleaved zygote (Figure 7L). They are mostly inherited by the D quadrant and detected in all teloblast lineages (mesodermal and ectodermal). Expression of this gene is observed in the descendants of the animal micromeres as well as in the bandlets of the blast cells during stages 1–5 (Figure 7M–O). During the next stages, 6–8, the levels of the transcripts are decreased in anteroposterior progression. The residual expression is observed at the caudal end of the embryo. *Eco-pl10-2* expression persists in the posterior growth zone at the juvenile worm stage.

#### 3.3.3. *Piwi* Homolog Expression during Development of the Annelid *E. coronatus*

**Figure 8:**
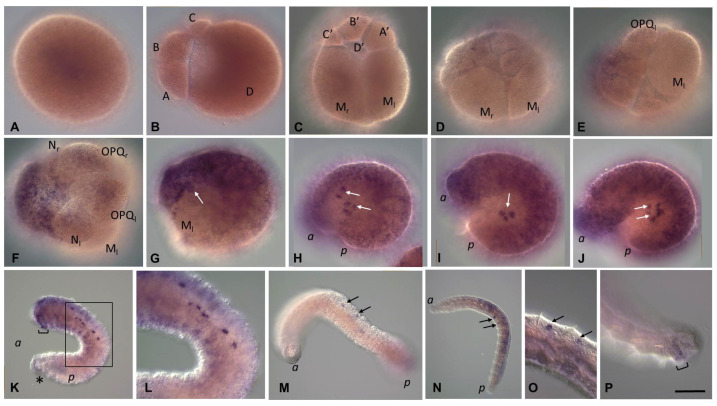
*Eco-piwi1A* expression patterns during stage 1 (**A**–**C**), stage 2 (**D**–**G**), stage 3 (**H**), stage 4 (**I**,**J**), stage 5 (**K**,**L**), stage 7 (**M**), and in juvenile worms (**N**–**P**). Lateral view, except (**B**) (view from animal pole), (**C**) (ventral view), (**D**) (ventral view from anterior), and (**F**) (dorsolateral view). (**A**) *Eco-piwi1A mRNA* is found in a zygote. (**B**) Four-cell embryo. (**C**) Transcripts are detected at low levels in mesodermal teloblasts before proteloblast formation. (**D**–**F**) Expression signal reappears in the N and OPQ cells and occurs in the descendants of micromeres and in blast cells at the animal pole. (**G**) At the end of stage 2, a few *Eco-piwi1A*-positive cells become visible at the anterior pole of the embryo (presumptive PGCs). (**H**–**J**) Strong *Eco-piwi1A* expression in the elongating germband. PGCs become located more ventrally. (**K**) Transcript levels in the germband cells gradually disappear in anteroposterior progression. PGCs become migrating. (**L**) Enlarged view of the boxed region shown in (**K**). (**M**) PGCs migrate to the gonadal segments. (**N**–**P**) *Eco-piwi1A* expression persists in the putative germline cells (**N**,**O**) and in the posterior growth zone (**P**) at the juvenile stage. Arrows mark putative PGCs; an asterisk marks the mouth position; a square bracket marks the posterior growth zone; *a* and *p* mark the anterior and posterior ends, respectively. Scale bar, 50 µm for all panels except (**K**,**M**,**N**). Scale bar in (**K**) and (**M**), 75 µm; in (**N**), 170 µm.

*Eco-piwi1A mRNA* is found in zygotes (Figure 8A). However, during further cleavage, the signal almost completely fades. Transcripts of this gene cannot be detected by WMISH in any cells, except for mesodermal teloblasts (Figure 8B,C). At stage 2, a distinct expression signal reappears in the N and OPQ cells, as well as in the descendants of micromeres at the animal pole (Figure 8D–E). During meso- and ectoteloblast proliferation, *Eco-piwi1A* transcripts appear in their daughter cells that form the germband (Figure 8F,G). Simultaneously, they disappear in the ectoteloblasts and mesoteloblasts themselves. At the end of stage 2, a few *Eco-piwi1A*-positive cells become visible at the anterior pole of the embryo (presumptive PGCs) (Figure 8G). These deep cells are located anteriorly to mesoteloblasts in the form of two bilateral clusters, usually 2–3 cells each. During the germband elongation, they become located more ventrally, first in the middle part of the embryo, and then in its posterior part (Figure 8H–J). Strong expression of *Eco-piwi1A* in the germband persists during gastrulation and is homogeneous throughout the bandlets at stages 3–4. At these stages, transcripts are also found again in ectoteloblasts (Figure 8H–J). Later, transcript levels in ecto- and mesodermal cells gradually disappear in anteroposterior progression (Figure 8K). The number of deep *Eco-piwi1A*-positive cells increases slightly (to 6–7 on each side), and the cells become migrating at the end of stage 4 (Figure 8K–M). At stage 7, *Eco-piwi1A mRNA* is detected in cells of the posterior growth zone and in the putative germline cells (Figure 8M), which are found in future gonadal segments 10 and 11. This pattern of expression remains in juvenile worms (Figure 8N–P).

**Figure 9:**
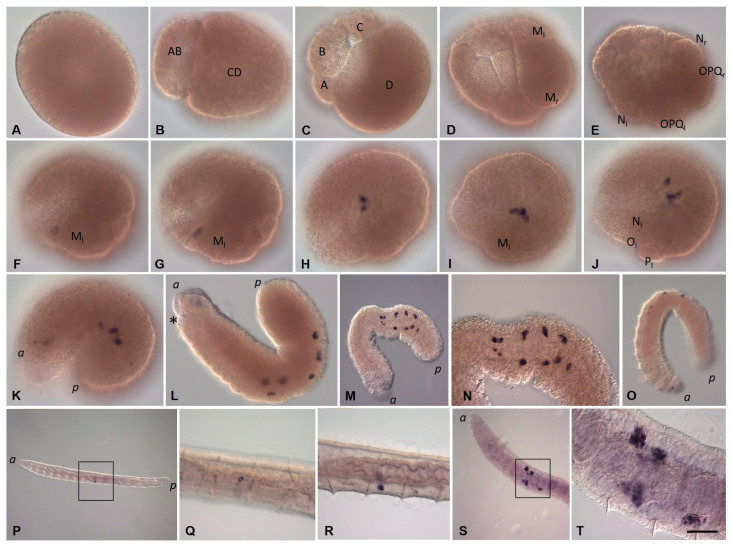
*Eco-piwi1B* expression patterns during stage 1 (**A**–**E**), stage 2 (**F**,**G**), stage 3 (**H**,**I**), stage 4 (**J**,**K**), stage 5 (**K**,**L**), stage 6 (**M**,**N**), stage 7 (**O**), and in juvenile worms (**P**–**T**). Lateral view (**A**,**F**–**L**,**O**,**S**,**T**), ventrolateral view (**D**,**M**,**N**,**P**–**R**), and dorsal view (**E**). (**A**–**E**) At stage 1, no transcripts are detected in oocytes or any blastomeres. (**A**) Zygote. (**B**) Two-cell embryo. (**C**) Four-cell embryo. (**D**) Mesoteloblasts. (**E**) OPQ proteloblasts and N ectoteloblasts. (**F**,**G**) At the end of stage 2, a few *Eco-piwi1B*-positive cells become visible (presumptive PGCs), different focal plane. (**H**–**J**) Clusters of the presumptive PGCs move during the germband elongation. (**K**–**O**) The number of putative PGCs increases. PGCs migrate. (**P**–**T**) *Eco-piwi1B* expression persists in putative germline cells, which are located in gonadal segments 10 and 11. (**Q**,**R**) Enlarged view of the boxed region shown in (**P**), different focal plane. (**S**,**T**) A 1-week-old juvenile animal. (**T**) Enlarged view of the boxed region shown in (**S**). The asterisk marks the mouth position; *a* and *p* mark the anterior and posterior ends, respectively. Scale bar, 50 µm for all panels except (**M**,**O**,**P**,**S**). Scale bar in (**M**,**O**), 75 µm; in (**P**,**S**), 170 µm.

In contrast to *Eco-piwi1A*, there is no evidence of *Eco-piwi1B* gene expression in oocytes or any blastomeres at stage 1 (Figure 9A–E). The first signs of this gene expression are detected in two bilateral clusters of putative PGCs at the end of stage 2 (Figure 9F,G). Transcript levels are initially low, but staining in these two clusters of cells becomes stronger as the clusters move during the germband elongation (Figure 9H–K). The number of putative PGCs increases to six in each cluster (Figure 9I). The cells begin migrating at the end of the stage 4 (Figure 9J). At stage 7, *Eco-piwi1B* expression persists in putative germline cells, which are located in gonadal segments 10 and 11 (Figure 9O). In juvenile worms, the number of such cells starts to increase (Figure 9S,T). *Eco-piwi1B* expression in cells of the posterior growth zone is not detected at any stage of development (Figure 9).

#### 3.3.4. *Nanos* Homolog Expression during Development of the Annelid *E. coronatus*

**Figure 10:**
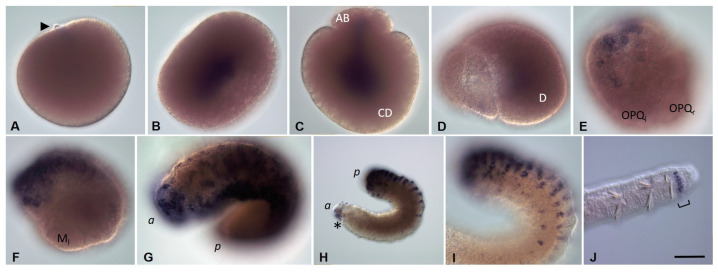
*Eco-nanos1* expression patterns during stage 1 (**A**–**E**), stage 2 (**F**), stage 4 (**G**), stage 5 (**H**,**I**), and in juvenile worms (**J**). Lateral view, except (**C**) (view from animal pole) and (**E**) (dorsolateral view). (**A**) End of the second meiotic division. (**B**) Zygote. (**C**) Two-cell embryo. (**D**) Four-cell embryo. (**E**) Expression is detected in several animal micromeres and in the first n-blast cells. (**F**) Expression in all superficial cells of the prospective anterior end of the embryo, including the cells of the germband. (**G**,**H**) The domain of expression expands posteriorly but begins to disappear gradually in the anterior half of the embryo, except for the developing stomodeum, brain, and ventral nerve cord. (**H**,**I**) The expression pattern becomes metameric. (**I**) An enlarged view of the embryo is shown in (**H**). (**J**) Expression in the posterior growth zone. The arrowhead marks the second polar body; the asterisk marks the mouth position; the square bracket marks the posterior growth zone; *a* and *p* mark the anterior and posterior ends, respectively. Scale bar, 50 µm for all panels except (**H**,**J**). Scale bar in (**H**,**J**), 75 µm.

*Eco-nanos1 mRNA* is accumulated in the perinuclear cytoplasm during zygote formation and is then segregated into all blastomeres of the two- and four-cell embryos (Figure 10A–D). The D blastomere inherits most of the transcripts. However, the mRNA quickly disappears, including in mesodermal and ectodermal teloblasts. At the end of stage 1, *Eco-nanos1* expression is detected in several animal micromeres and in the first n-blast cells (Figure 10E). Later, it increases and can be found in all superficial cells of the prospective anterior end of the embryo, including the cells of the germband (Figure 10F). During stages 3 and 4, the domain of expression expands posteriorly but begins to disappear gradually in the anterior half of the embryo, except for the developing stomodeum and brain (Figure 10G). During *E. coronatus* development, a particularly high level of *Eco-nanos1* transcripts is observed in the leading marginal cells that overgrow the endoderm and form the ventral nerve cord. At stage 5, the signal is still observed in the brain and foregut anlages, as well as on the ventral side of the middle and posterior part of the embryo (Figure 10H). In the posterior part of the embryo, expression persists on the dorsal side until the process of overgrowing the endoderm is completed. The expression pattern becomes metameric (Figure 10H,I). Expression gradually disappears, except for the cells at the posterior end, where a growth zone is formed. In juveniles, transcripts are found in the posterior growth zone (Figure 10J).

**Figure 11:**
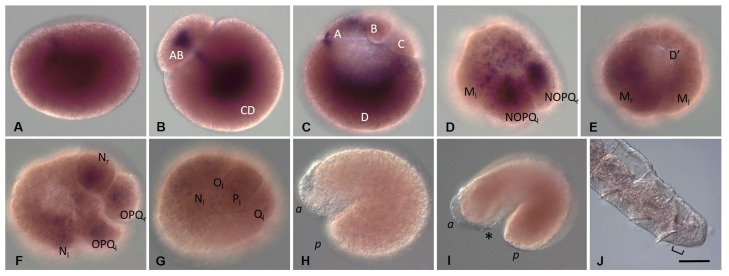
*Eco-nanos2* expression patterns during stage 1 (**A**–**F**), stage 2 (**G**), stage 4 (**H**,**I**), and in juvenile worms (**J**). (**A**) Zygote. (**B**) Two-cell embryo, view from animal pole. (**C**) Four-cell embryo, view from animal pole. (**D**–**F**) Expression in animal micromeres and the cells of teloblast lineage (mesodermal and ectodermal), dorsolateral (**D**), ventral (**E**), or dorsal view (**F**). (**G**) At the end of stage 2, transcripts disappear from all cells of the teloblast lineage. (**H**–**J**) *Eco-nanos2 mRNA* is not detected by WMISH. The asterisk marks the mouth position; the square bracket marks the posterior growth zone; *a* and *p* mark the anterior and posterior ends, respectively. Scale bar, 50 µm for all panels.

Transcripts of the second *Nanos* ortholog, *Eco-nanos2*, are found in the cytoplasm of zygotes (Figure 11A). At the stage of two and four blastomeres, all cells contain the mRNA of this gene (Figure 11B,C). Later, however, *Eco-nanos2* expression is observed only in mesoteloblasts and proteloblasts, and then in the N and OPQ cells (Figure 11D–F). At the end of stage 2, transcripts disappear from all cells of the teloblast lineage (Figure 11G). During the next developmental stages, including the juvenile worm stage, *Eco-nanos2 mRNA* is not detected by WMISH (Figure 11H–J).

#### 3.3.5. Eco-myc Embryonic and Juvenile Expression Patterns

**Figure 12:**
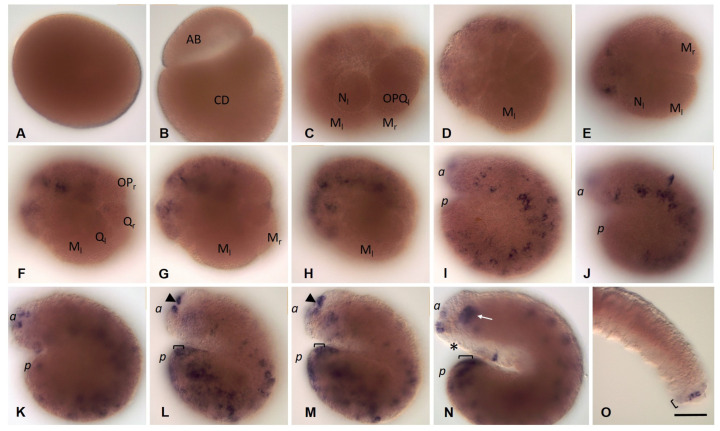
*Eco-myc* expression patterns during stage 1 (**A**–**C**), stage 2 (**D**,**E**), stage 3 (**F**–**H**), stage 4 (**I**–**L**), stage 5 (**K**), and in juvenile worms (**O**). Lateral view, except (**B**) (view from animal pole) and (**D**) (dorsal view). (**A**) Zygote. (**B**) Two-cell embryo. (**C**) OPQ proteloblasts and N ectoteloblasts. (**D**–**K**) Expression in animal micromeres and blast cells. (**K**–**N**) Expression in the brain and foregut primordia. (**L**–**O**) Expression in the posterior growth zone. The arrowhead marks the brain primordium; the arrow marks the pharynx; the asterisk marks the mouth position; the square bracket marks the posterior growth zone; *a* and *p* mark the anterior and posterior ends, respectively. Scale bar, 50 µm for all panels except (**O**). Scale bar in (**O**), 75 µm.

At stage 1, expression of *Eco-myc* gene is not detected in either oocytes or blastomeres (Figure 12A–C). At the beginning of stage 2, *Eco-myc* transcripts are shown at low levels in a few animal micromeres and individual cells of the germband (Figure 12D,E). Expression is particularly prominent in the n-blast cells (Figure 12H–J). At stage 4, *Eco-myc mRNA* is found in the brain and foregut primordia. It persists during the next two days (Figure 12K–N). At stage 4, expression of this gene is detected in teloblasts (mesodermal and ectodermal) (Figure 12K). Later, transcripts disappear in anteroposterior progression. In juveniles, expression is found in the posterior growth zone (Figure 12O).

#### 3.3.6. *Pumilio* Homolog Expression during Development of the Annelid *E. coronatus*

**Figure 13:**
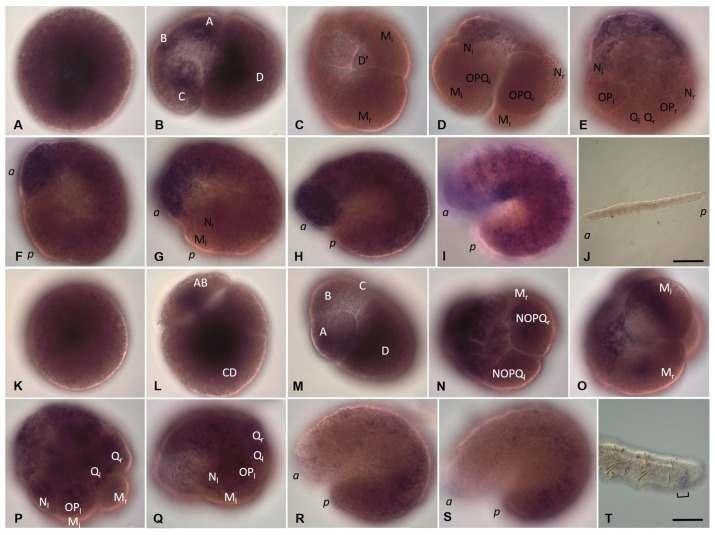
*Eco-pumilio2* (**A**–**J**) and *Eco-pumilio3* (**K**–**T**) expression patterns during stage 1 (**A**–**D**,**K**–**O**), stage 2 (**E**,**F**,**P**,**Q**), stage 3 (**H**,**R**), stage 4 (**I**,**S**), and in juvenile worms (**J**,**T**). Lateral view, except (**C**,**O**) (ventral view), (**D**,**N**,**P**) (dorsal view), and (**L**) (view from animal pole). (**A**,**K**) Zygote. (**L**) Two-cell embryo. (**B**,**M**) Four-cell embryo. (**C**–**E**) *Eco-pumilio2* is not detected in teloblasts during their formation. (**E**–**I**) *Eco-pumilio2* expression in the blast cells forming the germband. (**J**) There is no evidence of *Eco-pumilio2* gene expression in juvenile worms. (**N**–**Q**) *Eco-pumilio3* expression becomes restricted to the teloblast lineages. (**R**,**S**) *Eco-pumilio3* expression begins to disappear in anteroposterior progression. (**T**) Expression in the posterior growth zone. The square bracket marks the posterior growth zone; *a* and *p* mark the anterior and posterior ends, respectively. Scale bar, 50 µm for all panels except (**J**,**T**). Scale bar in (**J**), 170 µm; in (**T**), 75 µm.

Transcripts of both identified *Pumilio* orthologs, *Eco-pumilio2* and *Eco-pumilio3*, are found in the cytoplasm of zygotes and all blastomeres at the two- and four-cell embryo stages (Figure 13A,B,K–M).

*Eco-pumilio2 mRNA* disappears during the next rounds of cleavage (Figure 13C). The levels of *Eco-pumilio2* transcripts peak again in the blast cells forming the germband (Figure 13D–H). Expression of this gene is detected in ectodermal teloblasts during stages 3–5 (Figure 13G). Within the germband, expression shows a metameric pattern at the end of stage 4 (Figure 13I) and then decreases in anteroposterior progression during the next two days. There is no evidence of *Eco-pumilio2* gene expression in juvenile worms (Figure 13J).

*Eco-pumilio3* expression is detected in most if not all blastomeres of all four embryonic quadrants during the stage 1 (Figure 13L–O). Later, it becomes restricted to the teloblast lineages, including the blast cells forming the germband (Figure 13P). At the end of stage 4, expression begins to disappear in anteroposterior progression (Figure 13R,S). In juveniles, expression is found in the posterior growth zone (Figure 13T).

#### 3.3.7. *Tudor*, *Boule*, and *Bruno* homolog expression during development of the annelid *E. coronatus*

**Figure 14:**
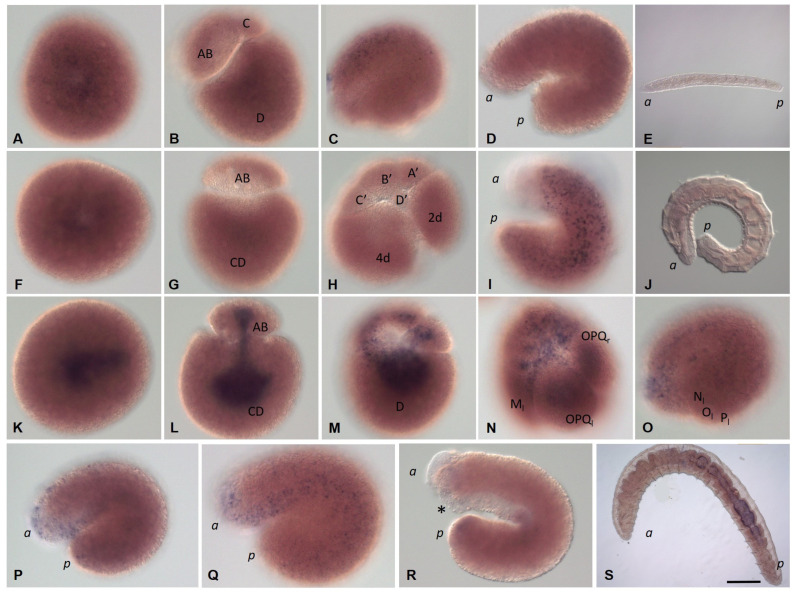
*Eco-tudor1* (**A**–**E**), *Eco-tudor2* (**F**–**J**), and *Eco-tudor3* (**K**–**S**) expression patterns during stage 1 (**A**,**B**,**F**–**D** and **K**–**N**), stage 3 (**C**,**O**), stage 4 (**D**,**I**,**P**,**Q**), stage 5 (**I**,**R**), and in juvenile worms (**E**,**J**,**S**). Lateral view, except **G**,**L,** and **M** (view from animal pole), **H** (ventral view), and **N** (dorsal view). (**A**,**F**,**K**) Zygote. (**G**,**L**) Two-cell embryo. (**B**,**M**) Four-cell embryo. (**C**) *Eco-tudor1* transcripts are detected at low levels in germband cells. (**D**) Later, *Eco-tudor1* expression gradually disappears. (**I**) *Eco-tudor2* expression persists in individual ectodermal cells of the germband until stage 5. (**N**) *Eco-tudor3* expression in cells of the teloblast lineage, including blast cells and meso- and ectoteloblasts. (**O**–**R**) Weak *Eco-tudor3* expression persists in the ectodermal cells of the anterior end of the embryo and the germband, including the cells that form the ventral nerve cord. (**E**,**J**,**S**) In juveniles, no expression of identified *Tudor* homologs is detected by WMISH. In (**S**) there is an unspecific staining in the gut lumen. The asterisk marks the mouth position; *a* and *p* mark the anterior and posterior ends, respectively. Scale bar, 50 µm for all panels except (**E**,**J**,**T**). Scale bar in (**E**), 170 µm; in (**J**,**T**), 75 µm.

Transcripts of all three cloned *Tudor* orthologs, *Eco-tudor1*, *Eco-tudor2*, and *Eco-tudor3*, are found in oocytes, but the level of *Eco-tudor3* transcripts is most likely significantly higher than the levels of other orthologs (Figure 14A,F,K). mRNAs of *Eco-tudor1* and *Eco-tudor2* genes disappear during blastomere cleavage (Figure 14B,G,H). At stage 3, transcripts of both genes are detected again at low levels in germband cells (Figure 14C). Later, *Eco-tudor1* expression weakens and gradually disappears (Figure 14D). *Eco-tudor2* expression persists in individual ectodermal cells of the germband until stage 5 (Figure 14I), and then it is no longer detected. In juveniles, expression of both genes could not be demonstrated using WMISH (Figure 14E,J).

High levels of *Eco-tudor3 mRNA* remain in cells of the teloblast lineage, including blast cells and meso- and ectoteloblasts (Figure 14L–N). At stage 2, the level of transcripts of this gene decreases rapidly. However, weak *Eco-tudor3* expression persists in the ectodermal cells of the anterior end of the embryo and the germband, including the cells that form the ventral nerve cord, during the next two stages of development (Figure 14O–R). Juvenile worms show no evidence of *Eco-tudor3* gene expression (Figure 14S).

Although the mRNA of both *Boule* orthologs, *Eco-boule1* and *Eco-boule2*, is detected in oocytes, it disappears rapidly during the first rounds of cleavage (Supplementary Materials, Figure S9). At stage 3, a weak diffuse expression of both genes is found again in the descendants of the animal micromeres and germband cells. At stage 4, *Eco-boule1* expression becomes particularly prominent in cells of the animal pole and the ventral side of the anterior part of the embryo, where the ventral nerve cord begins to form. At this stage, *Eco-boule2* expression in superficial cells is very weak, except for a domain in the most anterior part of the embryo. At stage 5, both transcripts disappear rapidly and are no longer detectable, including the juvenile worm stage.

**Figure 15:**
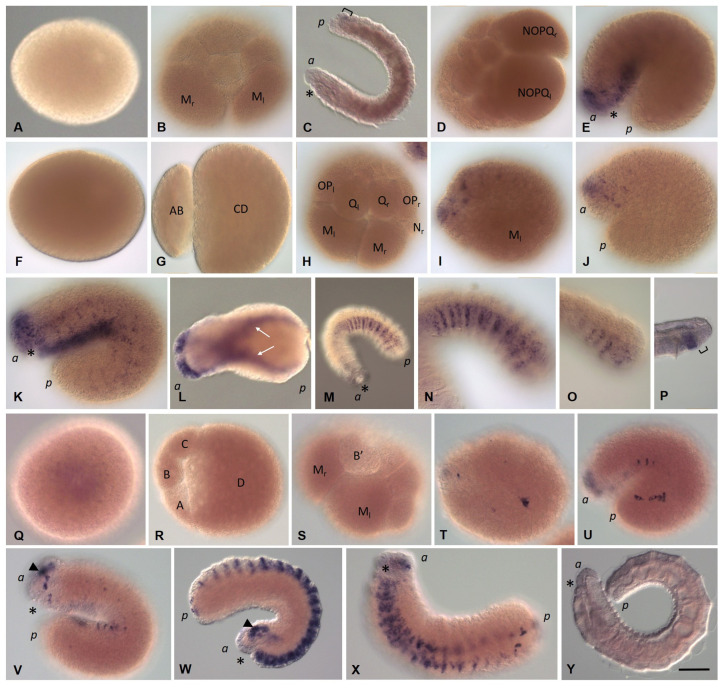
*Eco-bruno1* (**A**–**C**), *Eco-bruno2* (**D**,**E**), *Eco-bruno3* (**F**–**P**), and *Eco-bruno4* (**Q**–**Y**) expression patterns during stage 1 (**A**,**B**,**D**,**F**–**H**,**Q**–**S**), stage 2 (**H**), stage 3 (**I**,**J**,**T**), stage 4 (**E**,**J**–**L**,**U**), stage 5 (**M**,**V**,**W**), stage 6 (**N**,**O**,**X**), and in juvenile worms (**C**,**P**,**Y**). Lateral view, except (**B**) and (**X**) (ventral view), (**D**,**H**,**L**) (dorsal view), (**G**,**R**) (view from animal pole), and (**S**) (ventrocaudal view). (**A**,**F**,**Q**) Zygote. (**G**) Two-cell embryo. (**R**) Four-cell embryo. (**C**) *Eco-bruno1* expression in the posterior growth zone. (**B**,**D**,**H**,**S**) No expression of identified *Bruno* homologs is detected at the beginning of the germband formation. (**E**,**I**–**L**) *Eco-bruno2* and *Eco-bruno3* expression in the descendants of the animal micromeres and in the germband cells during gastrulation. (**L**) Deep focal plane from the dorsal side. Arrows show the *Eco-bruno3*-expressing marginal cells that overgrow the endoderm and form the ventral nerve cord. (**M**,**N**) Expression of *Eco-bruno3* gene persists along the entire length of the germ band but shows metameric pattern. (**P**) In juveniles, *Eco-bruno3* expression in the ventral domain is located anterior to the posterior growth zone. (**Q**–**S**) *Eco-bruno4* transcripts are found in oocytes, but mRNA of this gene disappears during blastomere cleavage. (**T**) At stage 3, *Eco-bruno4* expression occurs in a few cells of the germband. (**U**–**X**) *Eco-bruno4* expression in the developing brain, pharynx, and ventral nerve cord. (**Y**) *Eco-bruno4* expression is no longer detectable at the juvenile worm stage. The asterisk marks the mouth position; the arrowhead marks the developing brain; the square bracket marks the posterior growth zone; *a* and *p* mark the anterior and posterior ends, respectively. Scale bar, 50 µm for all panels except (**C**,**L**,**M**,**P**,**Y**). Scale bar in (**C**,**M**,**P**,**Y**), 75 µm; in (**L**), 60 µm.

Transcripts of the *Eco-bruno1*, *Eco-bruno2*, and *Eco-bruno3* genes are not detected in oocytes and embryos during the first two days of development (Figure 15A,B,D,F–H). Moreover, *Eco-bruno1 mRNA* is shown only at the very last stages of development and in juveniles. It appears in the posterior growth zone (Figure 15C). In contrast, the expression of *Eco-bruno2* and *Eco-bruno3* is detected again at stage 3. Transcripts of these genes mark the descendants of the animal micromeres and anterior blast cells (Figure 15E,I,J). Expression becomes particularly prominent in the anterior part of the developing ventral nerve cord. At stage 5, *Eco-bruno2* transcript levels begin to decline in anteroposterior progression. At stage 6 and later, including the juvenile animal stage, *Eco-bruno2 mRNA* is no longer detectable. In contrast, the expression of *Eco-bruno3* gene persists along the entire length of the germ band but shows metameric pattern (Figure 15K–O). Later, *Eco-bruno3* transcript levels also begin to decline in anteroposterior progression. In juveniles, *Eco-bruno3* expression is observed in the ventral domain located anterior to the posterior growth zone (Figure 15P). Perhaps, in this part of the posterior end of the worm, the formation of a new part of the ventral nerve cord occurs within the young segments.

*Eco-bruno4* transcripts are found in oocytes, but mRNA of this gene disappears during blastomere cleavage (Figure 15Q–S). At stage 3, transcripts are again detected in a few cells of the germband (Figure 15T). Next, the number of *Eco-bruno4*-positive cells increases markedly. All of them are ectodermal cells located ventrally or at the anterior end of the embryo (Figure 15U,V). At stage 5, when the main processes of formation of the central nervous system and foregut occur, *Eco-bruno4* expression becomes particularly prominent. In cells of the developing brain, pharynx, and ventral nerve cord, the level of transcripts becomes noticeably higher (Figure 15W,X). After the formation of the central nervous system is completed, *Eco-bruno4* expression is no longer detectable, including the juvenile worm stage (Figure 15Y).

## 4. Discussion

To answer the question of the possible origin of the PGCs and to begin elucidating mechanisms by which the germ line is specified in *Enchytraeus coronatus* embryos, we re-described the early embryogenesis, identified twenty germline/multipotency genes, homologs of *Vasa*, *PL10*, *Piwi*, *Nanos*, *Myc*, *Pumilio*, *Tudor*, *Boule*, and *Bruno*, and examined their developmental patterns using WMISH.

### 4.1. Early Embryogenesis and Possible Origin of PGCs in E. coronatus

In *E. coronatus*, fertilization and oocyte maturation occur in a cocoon. Zygotes are yolk-rich and variable in shape and size. They are relatively small in size, with a diameter of only 150–160 µm. Clear morphological signs of ooplasmic segregation, as described for other annelids [71,72], including accumulation of pole plasm (teloplasm), characteristic of *Tubifex* and leeches [73], are not observed. However, the egg changes shape several times during the second meiotic division, and the largest cell of the embryo, D blastomere, inherits most of the yolk-free cytoplasm and numerous maternal gene products (see below).

*E. coronatus* exhibits a modified version of spiral cleavage, although it is less modified than in the leech and other clitellates with the eggs less rich in yolk [49,50]. Therefore, the basic spiral cleavage sequence of spindle orientations and cleavage planes is retained but is accompanied by a reduction in the number of cell divisions leading to the blastula. The blastula is a stereoblastula, and we did not observe blastocoel formation, in contrast to [64]. As in other annelids, the D blastomere gives rise to the two somatoblasts. The first somatoblast (2d cell) and the second somatoblast (4d cell) finally differentiate into the body ectoderm and mesoderm, respectively.

In contrast to many annelids, in *E. coronatus*, the 2d cell does not show three rounds of highly unequal divisions before forming the proteloblast NOPQ by bilateral division. Although it undergoes a highly unequal division, giving off a smaller daughter cell (2d^1^) anteriorly, daughter cells of which form a single cell line that separates bilaterally located ectodermal germbands, as described in [64].

In *E. coronatus*, the mesoteloblasts are distinctively larger than the NOPQ cells. As in *Tubifex* and in the leech [49,50,74], after their appearance, both NOPQs divide twice by highly unequal divisions cutting off the smaller cells anteriorly. Proteloblasts divide synchronously, finally forming four ectoteloblasts (N, O, P, and Q) on either side of the embryo. The ectoteloblasts generate the ectoderm and its neural derivatives by teloblastic divisions. Teloblastic divisions are typical for mesoteloblasts, which are daughter cells of the second somatoblast.

First, mesoteloblasts undergo two rounds of highly unequal divisions, giving off the smaller daughter cells anteriorly. These small cells exhibit a high nucleo-cytoplasmic ratio. According to our data, including the expression of germ cell markers, these cells give rise to PGCs (see below). Mesoteloblasts then form two rows of m-blast cells. These m-blast cells are much larger than the products of the first two divisions and divide almost equally. Thus, in *E. coronatus*, as in other tubificids and enchytraeids [49,50,75], the PGCs are derived from a pair of small cells, descendants of 4d, and are closely associated with the presumptive mesoderm at the posterior end of the blastula.

### 4.2. Germ Line/Multipotency Genes in E. coronatus

The search for homologs of known members of germline multipotency program genes, GMP, in the *E. coronatus* transcriptome led to the discovery of numerous duplication events for most of the genes of interest. This observation was further validated through molecular cloning, resulting in a comprehensive catalog of homologs within the GMP group. As a result, two paralogs for *Vasa*, *PL10*, *Piwi*, *Nanos*, *Pumilio*, and *Boule*, three paralogs for *Tudor*, four paralogs for *Bruno*, and a single copy of *Myc* were identified. Except for *Eco-vasa1*, *Eco-pl10-1*, *Eco-pl10-2*, *Eco-tudor1*, *Eco-tudor2*, and *Eco-tudor3*, all amplified fragments include complete CDS, and 5′ and 3′ UTR.

To affirm the success of the cloning procedure and further explore the evolutionary history of these genes, a phylogenetic analysis was conducted using the Bayesian inference approach. The resulting trees demonstrate several notable patterns (Supplementary Materials, Figures S1–S8).

As anticipated, the majority of the studied genes from *E. coronatus* formed clusters with orthologs from other members of the Clitellata group, which encompasses oligochaetes and leeches. This pattern was observed for the cloned homologs of *Boule*, *Nanos*, *Vasa*, *Piwi*, *Pumilio*, and *Myc*.

**Figure 16:**
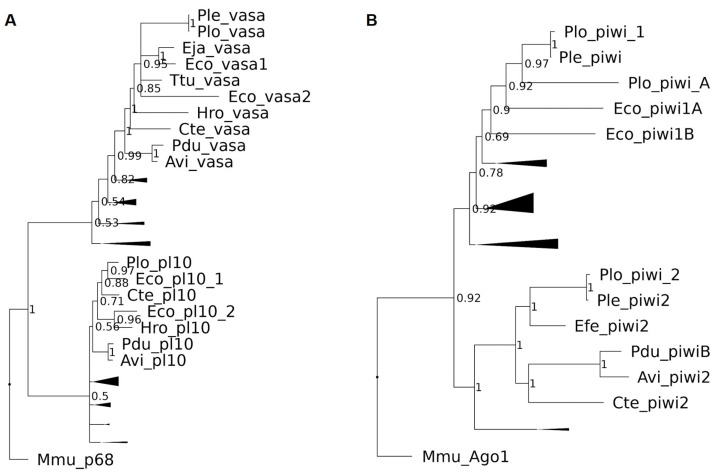
Evolutionary relationships between annelid *Vasa, Pl10* (**A**) and *Piwi* (**B**) homologs. The trees are simplified and scaled to highlight clades housing homologs from various annelid species. Non-relevant clades are collapsed for clarity. Supplementary materials contain the full, detailed versions of the trees. Abbreviations: Avi—*Alitta virens*, Cte—*Capitella teleta*, Eco—*Enchytraeus coronatus*, Efe—*Eisenia fetida*, Eja—*Enchytraeus japonensis*, Hro—*Helobdella robusta*, Pdu—*Platynereis dumerilii*, Ple—*Pristina leidyi*, Plo—*Pristina longiseta*, Ttu—*Tubifex tubifex*.

Interestingly, both discovered *Piwi* homologs, *Eco-piwi1A* and *Eco-piwi1B*, were found to belong to the *Piwi1* clade, contrary to another oligochaete member, *Pristina longiseta*, for which distinct *Piwi1* and *Piwi2* homologs were found in a recent study [9]. Both *Eco-piwi 1A* and *Eco-piwi1B* are oligochaete-specific and grouped with orthologs from the Naididae family species *P. leydii* and *P. longiseta* (Figure 16).

Another interesting pattern can be seen in *Pl10* gene evolution. We can notice that *Eco-pl10-2* is grouped with *H. robusta Pl10*, while the clade with *Eco-pl10-1* not only includes the oligochaete *P. longiseta* but also the polychaete *C. teleta*, a species outside the Clitellata group. This indicates a more ancient origin of *Pl10* duplication in annelids (Figure 16).

The phylogeny of *Tudor* and *Bruno* genes presented a unique challenge due to their complex structures with a variable number of domains. Additionally, the high diversity of these genes, as demonstrated in previous studies [76,77], added complexity to their analysis. In the case of the *Bruno* genes, four homologs were found in the *E. coronatus* transcriptome. Evolutionary analysis revealed that two of these homologs grouped with CUGBP-Elav-like family members 1 and 2. *Eco-bruno2* is clustered with homologous sequences from the leech *H. robusta*, whereas *Eco-bruno1* is grouped with genes from the polychaetes *P. dumerilii* and *C. teleta*. Both sets formed part of larger clades, which included vertebrate *CUGBP Elav-like family members 1 and 2*, consistent with previous phylogenetic studies [61,77]. The second pair of *E. coronatus Bruno* homologs is part of a big clade, containing *CUGBP-Elav-like family members 3 and 4* from invertebrate species. However, the situation is complicated by low support values, making it challenging to refine their evolution inside the annelid group. Duplicated homologs of the Tudor domain-containing proteins also demonstrated high structural variability. Our analysis resulted in a tree topology that generally aligned with previous studies [61,77], albeit with some exceptions, such as the position of *Trdr1* vertebrate genes and the grouping of *P. dumerilii* Tudor domain-containing proteins. In our case, two out of three cloned *E. coronatus* sequences fall into a clade, containing invertebrate homologs and vertebrate *Trdr6* sequences, where *Eco-tudor3* is grouped with annelid genes, including *P. dumerilii Tudor1* and *3*, while *Eco-tudor1* is more closely related to the last *P. dumerilii* homolog *Tudor2* and both are a sister group for vertebrate *Trdr6* sequences. The last cloned homolog, *Eco-tudor2*, formed a clade with *Branchiostoma lanceolatum* and vertebrate *Trdr1* sequences.

In conclusion, these findings provide valuable insights into the diversity, complexity, and evolutionary history of GMP genes in *E. coronatus*. It is essential to highlight that the sequencing of genes from other oligochaete species is imperative to address existing challenges in phylogenetic reconstruction and further advance our understanding of the evolutionary history of these genes.

### 4.3. Expression of the Germ Line/Multipotency Genes in E. coronatus

Transcripts of most of the identified GMP genes, except for *Eco-piwi1B*, *Eco-pl10-*1, Eco*-myc*, *Eco-bruno1*, *Eco-bruno2*, and *Eco-bruno3*, indicate maternal expression without specific asymmetric localization. They are detected in the yolk-free cytoplasm of oocytes. After the completion of the second meiotic division, they move from the animal pole to the center of the egg, accumulating in the perinuclear cytoplasm. Further distribution of mRNA of these genes in blastomeres of all four quadrants suggests the absence of the effect of possible ooplasmic segregation on the inheritance of the maternal transcript by specific blastomeres, although the largest blastomere (D) inherits most of the yolk-free cytoplasm and maternal mRNAs. Similarly, maternal transcripts of *Nanos*, *Piwi*, and *Vasa* homologs that are distributed broadly in early embryos have been shown for annelids (*Nanos* in *A. virens* [19], *Vasa* in *P. dumerilii* [25], *Vasa* and *Nanos* in *T. tubifex* [58,78]), *Vasa*, *Piwi*, and *Nanos* in *C. teleta* [6,79] and *H. robusta* [80,81], and mollusks (*Nanos* in *Tritia obsolete* [82], *Vasa*, *Pl10* and *Nanos* in *Haliotis asinine* [83]).

Later, transcripts of maternally expressed genes disappear in most cells, but *Eco-vasa1* mRNA is found at very low levels in the ectoteloblast lineage and animal micromeres. *Eco-vasa2 mRNA* is detected in the proteloblasts and ectoteloblasts, while the M-cells seem likely to be free of *Eco-vasa2* mRNA. High levels of *Eco-tudor3 mRNA* remain in cells of the teloblast lineage, including blast cells and meso- and ectoteloblast. Robust expression of most studied genes is detected in the ectodermal blast cells forming the germband at stages 2–4. Thus, it is without a doubt a zygotic phase of expression. A similar character of expression in the germband was reported for *Tubifex* (*Vasa*, *Nanos*) [58,78]) and *Helobdella* (*Vasa*, *Piwi*, and *Nanos*) [80,81]. In *E. coronatus*, as the germband elongates, the level of these gene expression in germband cells and descendants of micromeres gradually disappears, except for the most caudal region of the embryo, where the posterior growth zone develops. At stage 5, weak expression of *Eco-vasa1*, *Eco-pl10-1*, *Eco-nanos1*, *Eco-myc*, and *Eco-bruno4* can be detected in the pharynx anlage. *Eco-nanos1*, *Eco-pl10-1*, *Eco-myc*, *Eco-tudor3*, *Eco-boule1*, *Eco-bruno2*, *Eco-bruno3*, and *Eco-bruno4* are particularly prominently expressed in the developing ventral nerve cord and brain. Expression of these genes in the developing foregut and nervous system has been shown in a wide range of animals [6,9,15,19,84,85].

In contrast to the non-clitellate annelid *P. dumerilii* that shows expression of the homologs of almost all germline markers in the migrating PGCs at the stage of the nectochaete larva [61], in *E. coronatus*, only *Eco-vasa1*, *Eco-vasa2*, *Eco-piwi1A*, and *Eco-piwi1B* are expressed in the putative PGCs. *Eco-piwi1B* is the clearest marker because its expression is restricted to the germline cell, while expression of *Eco-vasa1*, *Eco-vasa2*, and *Eco-piwi1A* is observed in multiple tissues. In the germ line, the four genes show similar expression patterns to each other. At the end of stage 2, its transcripts appear in a few deep cells located anteriorly to mesoteloblasts in the form of two bilateral clusters. During the germband elongation, these clusters become located more ventrally, first in the middle part of the embryo, and then in its posterior part. The number of these marker-positive cells increases slightly (up to 6–7 on each side), and the cells begin migrating at the end of stage 4. At the end of embryonic development, the cells are found in future gonadal segments 10 and 11. In juvenile worms, transcripts of *Eco-vasa1*, *Eco-piwi1A*, and *Eco-piwi1B* persist, although *Eco-vasa2* expression in these cells disappears. Transcripts of both homologs of *Vasa (Ej-vlg1* and *Ej-vlg2*) and *Ej-piwi* were found in the testis, seminal vesicle, and ovary of mature *E. japonensis* worms. It was also found that germ-cell precursors are present in the prospective gonadal region even in asexually reproducing *E. japonensis* [7,12,57]. In contrast to nereids, *Alitta* and *Platynereis* [19,61], and the leech *Helobdella* [81], *Nanos* is not expressed in the putative PGCs in either *Tubifex* [78] or *Enchytraeus* (this study). Interestingly, in *Helobdella*, *Piwi* and *Vasa* homologs are expressed preferentially in female PGCs at a time when *Nanos* is expressed preferentially in male PGCs. According to our results, there are no differences in germline markers in PGCs during the embryonic development of *E. coronatus*. This suggests that most likely there is no heterogeneity in the population of primordial germ cells in *E. coronatus*. Moreover, in *Enchytraeus*, the putative PGCs derive from the mesoteloblasts during its first divisions, while in *Helobdella*, female and male PGC lineages are not segregated from mesoteloblasts until 6 and 8–18 rounds of M-cells divisions, respectively [81].

The behavior of the PGCs in nereids and enchytraeids is different. In *Alitta* and *Platynereis*, four PGCs originate by the first divisions of the mesoteloblasts. They remain mitotically inactive and clustered near the vegetal pole. At the nectochaeta larva stage, the PGCs start migrating anteriorly to a region posterior to the pharynx (primary gonad), where they form a single cluster, within which these cells will become proliferating in older juvenile worms [19,25,86]. In *E. coronatus*, two clusters of PGCs migrate during stages 2–4. The cells of these clusters show limited mitotic activity. At the end of stage 4, the clusters disappear and individual cells become migrating to the forming gonadal segments during the rest of embryonic development. The assumption made in the old literature (reviewed in [49,50]) that most presumptive germ cells degenerate during migration is not supported by our study using stem markers. In *E. coronatus*, the number of germ marker-positive cells remains stable (6–7 on either side of the embryo).

Although we have no data about proteins, both maternal and synthesized by maternal mRNA translations, the relatively late onset of germ cell marker gene expression in the PGCs (at the end of stage 2), suggests a possible combination of inherited cytoplasmic determinants, followed by inductive processes to determine which cells become the PGCs, as proposed for the annelid *P. dumerilii* [25]. Future studies shall involve the identification of mechanisms by which the germ line is specified in *E. coronatus* embryos.

## 5. Conclusions

Germline development and origin of the primordial germ cells (PGCs) are very variable and may occur across a range of developmental stages. The PGCs are segregated from specific cell lineages during cleavage, or they are specified by inductive interaction between cells at gastrulation stage. In establishing and maintaining a germ line, a conserved set of genes is involved that is also responsible for pluri- and multipotency and therefore has a broader role in governing “stemness” in both germline and somatic tissue. Our results suggest an early segregation of the PGCs in the embryos of the annelid *E. coronatus*. On the other hand, the relatively late onset of germ cell marker gene expression in the PGCs suggests a possible combination of inherited cytoplasmic determinants, followed by inductive processes to determine which cells become the PGCs.

## Data Availability

mRNA sequences of *Eco-vasa1*, *Eco-vasa2*, *Eco-pl10-1*, *Eco-pl10-2*, *Eco-piwi1A*, *Eco-piwi1B*, *Eco-nanos1*, *Eco-nanos2*, *Eco-myc*, *Eco-pumilio2*, *Eco-pumilio3*, *Eco-tudor1*, *Eco-tudor2*, *Eco-tudor3*, *Eco-boule1*, *Eco-boule2*, *Eco-bruno1*, *Eco-bruno2*, *Eco-bruno3*, and *Eco-bruno4* are deposited in GenBank with the accession numbers OR750672–OR750691.

## Supplementary Materials

The following supporting information can be downloaded at: https://www.mdpi.com/article/10.3390/biology12121508/s1, Table S1: Primer sequences used to clone fragments of the *Eco-vasa1*, *Eco-vasa2*, *Eco-pl10-1*, *Eco-pl10-2*, *Eco-piwi1A*, *Eco-piwi1B*, *Eco-nanos1*, *Eco-nanos2*, *Eco-myc*, *Eco-pumilio2*, *Eco-pumilio3*, *Eco-tudor1*, *Eco-tudor2*, *Eco-tudor3*, *Eco-boule1*, *Eco-boule2*, *Eco-bruno1*, *Eco-bruno2*, *Eco-bruno3*, and *Eco-bruno4* genes presented in the paper; Table S2: GenBank accession numbers for sequences used for Pl10 and Vasa amino acid alignments; Table S3: GenBank accession numbers for sequences used for Piwi amino acid alignments; Table S4:. GenBank accession numbers for sequences used for Nanos amino acid alignments; Table S5: GenBank accession numbers for sequences used for Myc amino acid alignments; Table S6: GenBank accession numbers for sequences used for Pumilio amino acid alignments; Table S7: GenBank accession numbers for sequences used for Tudor amino acid alignments; Table S8: GenBank accession numbers for sequences used for Boule amino acid alignments; Table S9: GenBank accession numbers for sequences used for RRM-domain protein Bruno amino acid alignments; Figure S1: Phylogenetic analysis of *Enchytraeus coronatus Vasa* and *Pl10* homologs; Figure S2: Phylogenetic analysis of *Enchytraeus coronatus Piwi* homologs; Figure S3: Phylogenetic analysis of *Enchytraeus coronatus Nanos* homologs; Figure S4: Phylogenetic analysis of *Enchytraeus coronatus Myc* homolog; Figure S5: Phylogenetic analysis of *Enchytraeus coronatus Pumilio* homologs; Figure S6: Phylogenetic analysis of *Enchytraeus coronatus Tudor* homologs; Figure S7: Phylogenetic analysis of *Enchytraeus coronatus Boule* homologs; Figure S8: Phylogenetic analysis of *Enchytraeus coronatus Bruno* homologs; Figure S9: *Eco-boule1* and *Eco-boule2* expression patterns.
