# Supplementary material for: Germ Line/Multipotency Genes Show Differential Expression during Embryonic Development of the Annelid Enchytraeus coronatus
Source: Biology (Basel). 2023 Dec 10;12(12):1508. doi: 10.3390/biology12121508 (PMC10740902; doi:10.3390/biology12121508)
Supplement: Supplementary file 1 [file biology-12-01508-s001.zip › biology-2751577-supplementary.pdf]

biology

MDPI

Article

Supplementary Materials for Germ Line/Multipotency Genes Show Differential Expression during Embryonic Development of the Annelid *Enchytraeus coronatus*

Roman P. Kostyuchenko *, Daria D. Nikanorova and Artem V. Amosov

Department of Embryology, St. Petersburg State University, Universitetskaya nab. 7-9, 199034 St. Petersburg, Russia; daria.nikanorova@mail.ru (D.D.N.), artem221199@mail.ru (A.V.A.)
* Correspondence: r.kostyuchenko@spbu.ru; Tel.: +7-812-3289453

Table S1. Primer sequences used to clone fragments of the Eco-vasa1, Eco-vasa2, Eco-pl10-1, Eco-pl10-2, Eco-piwi1A, Eco-piwi1B, Eco-nanos1, Eco-nanos2, Eco-myc, Eco-pumilio2, Eco-pumilio3, Eco-tudor1, Eco-tudor2, Eco-tudor3, Eco-boule1, Eco-boule2, Eco-bruno1, Eco-bruno2, Eco-bruno3, and Eco-bruno4 genes presented in the paper.

Eco-vasa1	forward	5′-CCCAACGCTGATAAGATGCCTGTC-3′
	reverse	5′-GGTTCTCGAGGAAGTTTGGAATCTC-3′
Eco-vasa2	forward	5′-GAGAAGATGGTAAGCCGAAGCCC-3′
	reverse	5′-CCACTCTGTATCATCGTTAGCAGC-3′
Eco-pl10-1	forward	5′-GGTGGAATCGTTTGAGGGAGGAGGAG-3′
	reverse	5′-CACCATTACCTCCCCCTCTCCTGTC-3′
Eco-pl10-2	forward	5′-CGAATGATTGGACTAAACCACTGCCC-3′
	reverse	5′-GGTAATCCCGCGAGGCAAATCCAGTG-3′
Eco-piwi1A	forward	5′-GGCGAGCACGTTCGAGAAGAAAG-3′
	reverse	5′-GGCTGGAGTCTAGTCGTGTCGTG-3′
Eco-piwi1B	forward	5′-GTTTGATGGGATGGGTCTGCTGTTC-3′
	reverse	5′-GAGGTTAGAGTTGTCGTAGATGAC-3′
Eco-nanos1	forward	5′-CTCGCCGATGTCTAGCCGTTCTTC-3′
	reverse	5′-CTCAGAGAATCGCCTGTGTTGTATG-3′
Eco-nanos2	forward	5′-CCCTCCACTAATTCTTCCACTAATCC-3′
	reverse	5′-CTACAGTTTGGCCCCAGTAGATTTAACC-3′
Eco-myc	forward	5′-CTCTAAACTGGCTGGAAGACGGAC-3′
	reverse	5′-CCCTGTCGAGCCTTGGCATTCCTC-3′
Eco-pumilio2	forward	5′-CAATACGCTCTGCCTACTGACCAAATAC-3′
	reverse	5′-CCGTATGGGTGCATGGCTAGGTTC-3′
Eco-pumilio3	forward	5′-CAATGCAGGATTTCAAGGAGGCG-3′
	reverse	5′-GCTGTAGGGTAGTGCTGCCACCTG-3′
Eco-tudor1	forward	5′-CCAACGCTAGACAGCTGGATAACC-3′
	reverse	5′-GTCACTGTAGCTCTGTACCAGGAC-3′
Eco-tudor2	forward	5′-CAAAGAGTTCAGCAGCAGTTGTGTG-3′
	reverse	5′-GTCCCCAGACACCGGTCTCAACTC-3′

Eco-tudor3	forward	5′-GATGACATCACTGCGGAGGCTGAG-3′
	reverse	5′-CTTTGAGGCCACATCTTCCCCATC-3′
Eco-boule1	forward	5′-CTACATTCTATCCGTCTAGACAGCAAAC-3′
	reverse	5′-CTGCATATCCACAGCTGATGACGG-3′
Eco-boule2	forward	5′-CTTCATTGTCGTCGCTGTGAGTCC-3′
	reverse	5′-CATCAGCTGAACATGACCGCCTG-3′
Eco-bruno1	forward	5′-GAACACTCAACCAATCACAGCAGAG-3′
	reverse	5′-CACAGACCGTTAGCGAGAGCAGC-3′
Eco-bruno2	forward	5′-CTGAATGCAGCAACCAGTGGGTCC-3′
	reverse	5′-GTAGAGGGTGGTATCAGACTTGGG-3′
Eco-bruno3	forward	5′-GTTCTGTTCCGTGCAACCGTATCG-3′
	reverse	5′-GTACGGTCGATTGGCGTCTTTAGG-3′
Eco-bruno4	forward	5′-GCATGTTTCATCGGCTGCGACTC-3′
	reverse	5′-CTCTTCAGTTGGACTTTCAACCTCTTC-3′

Table S2. GenBank accession numbers for sequences used for Pl10 and Vasa amino acid alignments.

**Phylum**	**Species**	**Gene name in tree**	**GenBank #**
Annelida	*Alitta virens*	Avi_pl10	AJW77404.1
	Capitella teleta	Cte_pl10	ELU14120.1
	Enchytraeus coronatus	Eco_pl10_1	OR750674
	Enchytraeus coronatus	Eco_pl10_2	OR750675
	Helobdella robusta	Hro_pl10	XP_009010523.1
	Platynereis dumerilii,	Pdu_pl10	CAJ15140.1
	Pristina longiseta	Plo_pl10	JX264564
	Alitta virens	Avi_vasa	AJW77403.1
	Capitella teleta	Cte_vasa	ELU18906.1
	Enchytraeus coronatus	Eco_vasa1	OR750672
	Enchytraeus coronatus	Eco_vasa2	OR750673
	Enchytraeus japonensis	Eja_vasa	BAF76795.1
	Helobdella robusta	Hro_vasa	XP_009030586.1
	Platynereis dumerilii	Pdu_vasa	CAJ38803.1
	Pristina leidyi	Ple_vasa	AIM52096.1
	Pristina longiseta	Plo_vasa	JX264563
	Tubifex tubifex	Ttu_vasa	BAD90012.1
Platyhelminthes	*Macrostomum lignano*	Mli_pl10	PAA56642.1
	Schistosoma japonicum	Sja_pl10	KAH8852518.1
Mollusca	*Crassostrea gigas*	Cgi_pl10	XP_011446932.2
	Patella vulgata	Pvu_pl10	XP_050413768.1
	Crassostrea gigas	Cgi_vasa	XP_034310873.1
	Lottia gigantea	Lgi_vasa	XP_009057808.1
	Patella vulgata	Pvu_vasa	XP_050411929.1

	Pecten maximus	Pma_vasa	P_033738811.1
Arthropoda	*Drosophila melanogaster*	Dme_belle	NP_536783.1
	Tribolium castaneum	Tca_belle	NP_001153721.1
	Drosophila melanogaster	Dme_vasa	CAA31405.1
	Tribolium castaneum	Tca_vasa	EFA07550.1
Echinodermata	*Strongylocentrotus purpuratus*	Spu_pl10	XP_030838128.1
	Lytechinus variegatus,	Lva_pl10	XP_041483221.1
	Patiria miniata	Pmi_pl10	XP_038050100.1
	Strongylocentrotus purpuratus	Spu_vasa	NP_001139665.1
	Lytechinus variegatus	Lva_vasa	XP_041475118.1
	Patiria miniata	Pmi_vasa	XP_038056422.1
Hemichordata	*Ptychodera flava*	Pfl_vasa	QOW95195.1
	Saccoglossus kowalevskii	Sko_vasa	XP_006825896.1
Chordata	*(outgroup) Mus musculus*	Mmu_p68	AAB53236.1
	Mus musculus	Mmu_pl10	NP_149068.1
	Danio rerio	Dre_pl10	AAH59794.1
	Mus musculus	Mmu_vasa	NP_001139357.1
	Danio rerio	Dre_vasa	AAI29276.1
	Branchiostoma floridae	Bfl_vasa	XP_035672419.1
Cnidaria	*Actinia tenebrosa*	Ate_pl10	XP_031554871.1
	Clytia hemisphaerica	Che_pl10	AFD28592.1
	Hydra vulgaris	Hvu_pl10	XP_047130892.1
	Nematostella vectensis	Nve_pl10	XP_048589030.1
	Actinia tenebrosa	Ate_vasa	XP_031562414.1
	Nematostella vectensis	Nve_vasa	AFP87471.1

Table S3. GenBank accession numbers for sequences used for Piwi amino acid alignments.

**Phylum**	**Species**	**Gene name in tree**	**GenBank #**
Annelida	*Aeolosoma viride*	Avr_piwi	AQV09901.1
	Alitta virens	Avi_piwi1	AJW77405.1
	Alitta virens	Avi_piwi2	AJW77406.1
	Capitella teleta	Cte_piwi1	DAA34898.1
	Capitella teleta	Cte_piwi2	ELU02261.1
	Eisenia fetida	Efe_piwi2	AUS83924.1
	Enchytraeus coronatus	Eco_piwi1A	OR750676
	Enchytraeus coronatus	Eco_piwi1B	OR750677
	Pristina leidyi	Ple_piwi	AIM52094.1
	Pristina leidyi	Ple_piwi2	AIM52095.1
	Pristina longiseta	Plo_piwi_A	JX264566
	Pristina longiseta	Plo_piwi_1	OR203685
	Pristina longiseta	Plo_piwi_2	JX264567

	Platynereis dumerilii	Pdu_piwi	CAJ28986.1
	Platynereis dumerilii	Pdu_piwiB	AZG02837.1
Mollusca	*Crassostrea virginica*	Cvi_piwi	XP_022338755.1
	Patella vulgata	Pvu_piwi	XP_050410271.1
Brachiopoda	*Lingula anatina*	Lan_piwi	XP_013405373.1
Arthropoda	*Drosophila melanogaster*	Dme_piwi	AAD08705.1
Echinodermata	*Strongylocentrotus purpuratus*	Spu_piwi	XP_030846820.1
	Lytechinus variegatus	Lva_piwi	XP_041456323.1
	Patiria miniata	Pmi_piwi	XP_038059144.1
Chordata	*(outgroup) Mus musculus*	Mmu_Ago1	NP_700452.2
	Mus musculus	Mmu_piwi1	NP_067286.1
	Mus musculus	Mmu_piwi2	NP_067283.1
	Mus musculus	Mmu_piwi4	BAF65667.1
	Danio rerio	Dre_piwi1	NP_899181.1
	Danio rerio	Dre_piwi2	ACF35261.1
	Branchiostoma floridae	Bfl_piwi1	XP_035691448.1
	Branchiostoma floridae	Bfl_piwi2	AGI95997.1
Cnidaria	*Actinia tenebrosa*	Ate_piwi	XP_031558248.1
	Hydra vulgaris	Hvu_piwi	AFQ20832.1
	Nematostella vectensis	Nve_piwi	ASW22510.1
	Nematostella vectensis	Nve_piwi2	ASW22511.1
	Podocoryna carnea,	Pca_piwi	AAS01181.1

Table S4. GenBank accession numbers for sequences used for Nanos amino acid alignments.

**Phylum**	**Species**	**Gene name in tree**	**GenBank #**
Annelida	*Alitta virens*	Avi_nanos	UFQ91327.1
	Capitella teleta	Cte_nanos	DAA06318.1
	Enchytraeus coronatus	Eco_nanos1	OR750678
	Enchytraeus coronatus	Eco_nanos2	OR750679
	Helobdella robusta	Hro_nanos	AAB63111.1
	Helobdella robusta	Hro_nanos1a	XP_009018920.1
	Paranais litoralis	Pli_nanos	ADE44351.1
	Platynereis dumerilii	Pdu_nanos	CAJ28985.1
	Pristina leidyi	Ple_nanos	ADE44350.1
	Tubifex tubifex	Ttu_nanos	BAQ21630.1
Mollusca	*Crassostrea virginica*	Cvi_nanos	XP_022345706.1
	Dreissena polymorpha	Dpo_nanos	XP_052227042.1
	Mizuhopecten yessoensis	Mye_nanos1	OWF49530.1
	Mizuhopecten yessoensis	Mye_nanos3	OWF55054.1
	Pecten maximus	Pma_nanos	XP_033757476.1
Echinodermata	*Lytechinus variegatus*	Lva_nanos	ANT70531.1

	Strongylocentrotus purpuratus	Spu_nanos1a	XP_001177221.2
	Strongylocentrotus purpuratus	Spu_nanos2	NP_001073023.1
Chordata	*Mus musculus*	Mmu_nanos1	NP_848508.2
	Mus musculus	Mmu_nanos2	NP_918953.2
	Mus musculus	Mmu_nanos3	NP_918948.1
	Xenopus laevis	Xla_nanos1	NP_001081503.1
	Xenopus tropicalis	Xtr_nanos3	XP_004919225.1
	Danio rerio	Dre_nanos1	NP_001292590.1
	Danio rerio	Dre_nanos2_2	XP_009300191.1
	Danio rerio	Dre_nanos3	NP_571953.1
Cnidaria	*Hydra vulgaris*	Hvu_nanos1	BAB01491.1
	Hydra vulgaris	Hvu_nanos2	BAB01492.1
	Nematostella vectensis	Nve_nanos1	AAY67907.1
	Nematostella vectensis	Nve_nanos2	AAY67908.1
Porifera	*Amphimedon queenslandica*	Aqu_nanos	XP_003384296.1
	Ephydatia muelleri	Emu_nanos	AJE59349.1

Table S5. GenBank accession numbers for sequences used for Myc amino acid alignments.

**Phylum**	**Species**	**Gene name in tree**	**GenBank #**
Annelida	*Capitella teleta*	Cte_myc	ATN39075.1
	Enchytraeus coronatus	Eco_myc	OR750680
	Helobdella robusta	Hro_myc	XP_009030581.1
	Owenia fusiformis	Ofu_myc	CAH1774206.1
	Platynereis dumerilii	Pdu_myc	AGS55451.1
Mollusca	*Crassostrea virginica*	Cvi_myc	XP_022318424.1
	Patella vulgata	Pvu_myc	XP_050400774.1
	Pecten maximus	Pma_myc	XP_033738863.1
Brachiopoda	*Lingula anatina*	Lan_myc	XP_013410679.1
Arthropoda	*Drosophila melanogaster*	Dme_myc	AAD00517.1
	Tribolium madens	Tma_myc	XP_044270248.1
	Daphnia pulex	Dpu_myc	EFX79343.1
	Limulus polyphemus	Lpo_myc	XP_013793685.1
Echinodermata	*Patiria miniata*	Pmi_myc	XP_038057555.1
	Strongylocentrotus purpuratus	Spu_myc	NP_999744.1
Hemichordata	*Saccoglossus kowalevskii*	Sko_myc	NP_001158444.1
Chordata	*Mus musculus*	Mmu_myc	NP_034979.3
	Danio rerio	Dre_mycA	NP_571487.2
	Danio rerio	Dre_mycB	NP_956466.1
	Branchiostoma floridae	Bfl_myc	XP_002609915.1
Cnidaria	*Actinia tenebrosa*	Ate_myc	XP_031551646.1
	Nematostella vectensis	Nve_myc	XP_001627619.2

Porifera	*Amphimedon queenslandica*	Aqu_myc	XP_011408076.1
	Halisarca dujardinii	Hdu_myc	QSX72220.1
Protista	*(outgroup) Monosiga brevicollis*	Mbr_myc	XP_001747958.1

Table S6. GenBank accession numbers for sequences used for Pumilio amino acid alignments.

**Phylum**	**Species**	**Gene name in tree**	**GenBank #**
Annelida	*Capitella teleta*	Cte_pum2	ELT98839.1
	Capitella teleta	Cte_pum3	ELU16136.1
	Enchytraeus coronatus	Eco_pum2	OR750681
	Enchytraeus coronatus	Eco_pum3	OR750682
	Helobdella robusta	Hro_pum2	XP_009016071.1
	Helobdella robusta	Hro_pum3	XP_009029294.1
	Owenia fusiformis	Ofu_pum3	CAH1774281.1
Mollusca	*Crassostrea gigas*	Cgi_pum2	XP_011422894.2
	Crassostrea gigas	Cgi_pum3	XP_034313950.1
	Lottia gigantea	Lgi_pum2	XP_009063233.1
	Lottia gigantea	Lgi_pum3	XP_009047841.1
	Patella vulgata	Pvu_pum3	XP_050403075.1
	Pecten maximus	Pma_pum3	XP_033737990.1
Brachiopoda	*Lingula anatina*	Lan_pum3	XP_013407952.1
Arthropoda	*Drosophila melanogaster*	Dme_pum	NP_731314.1
	Tribolium castaneum	Tca_pum	EFA12956.2
	Tribolium madens	Tma_pum2	XP_044266097.1
	Ixodes scapularis	Isc_pum2	XP_029825330.2
	Limulus polyphemus	Lpo_pum2	XP_013786730.1
Echinodermata	*Lytechinus variegatus*	Lva_pum2	XP_041466796.1
	Lytechinus variegatus	Lva_pum3	XP_041475303.1
	Strongylocentrotus purpuratus	Spu_pum2	XP_030830065.1
	Strongylocentrotus purpuratus	Spu_pum3	XP_030851927.1
	Patiria miniata	Pmi_pum2	XP_038051474.1
	Patiria miniata	Pmi_pum3	XP_038067463.1
Chordata	*Mus musculus*	Mmu_pum1	NP_109647.2
	Mus musculus	Mmu_pum2	XP_017170746.1
	Mus musculus	Mmu_pum3	NP_001390013.1
	Danio rerio	Dre_pum1	NP_001264048.1
	Danio rerio	Dre_pum2	NP_001096040.2
	Danio rerio	Dre_pum3	NP_001340777.1
Cnidaria	*Actinia tenebrosa*	Ate_pum2	XP_031566465.1
	Actinia tenebrosa	Ate_pum3	XP_031550523.1
	Hydra vulgaris	Hvu_pum2	XP_047145598.1
	Hydra vulgaris	Hvu_pum3	XP_002156370.2

	Nematostella vectensis	Nve_pum2	XP_032240209.1
	Nematostella vectensis	Nve_pum3	XP_001634611.1
Porifera	*Amphimedon queenslandica*	Aqu_pum2	XP_011402944.1
	Amphimedon queenslandica	Aqu_pum3	XP_003387437.1
Protista	*(outgroup) Monosiga brevicollis*	Mbr_pum	XP_001749459.1

Table S7. GenBank accession numbers for sequences used for Tudor amino acid alignments.

**Phylum**	**Species**	**Gene name in tree**	**GenBank #**
Annelida	*Capitella teleta*	Cte_Tdrd_ELU14575	ELU14575.1
	Enchytraeus coronatus	Eco_tudor1	OR750683
	Enchytraeus coronatus	Eco_tudor2	OR750684
	Enchytraeus coronatus	Eco_tudor3	OR750685
	Owenia fusiformis	Ofu_Tdrd_CAH1803308	CAH1803308.1
	Platynereis dumerilii	Pdu_Tdrd_1	CCK33035.1
	Platynereis dumerilii	Pdu_Tdrd_2	CCK33037.1
	Platynereis dumerilii	Pdu_Tdrd_3	CCK33036.1
Mollusca	*Crassostrea gigas*	Cgi_Tdrd_XP_011447159	XP_011447159.3
	Patella vulgata	Pvu_Tdrd_6	XP_050402777.1
	Pecten maximus	Pma_Tdrd_XP_033738269	XP_033738269.1
Brachiopoda	*Lingula anatina*	Lan_Tdrd_XP_013386808	XP_013386808.1
Arthropoda	*Drosophila melanogaster*	Dme_tudor	NP_476773.1
	Drosophila melanogaster	Dme_papi	NP_722773.1
	Drosophila melanogaster	Dme_tejas	NP_610950.2
	Limulus polyphemus	Lpo_Tdrd_1	XP_013776272.2
Echinodermata	*Strongylocentrotus purpuratus*	Spu_Tdrd_XP_782950	XP_782950.2
	Strongylocentrotus purpuratus	Spu_Tdrd_15_XP_780689	XP_780689.2
	Strongylocentrotus purpuratus	Spu_Tdrd_XP_797685	XP_797685.2
Hemichordata	*Saccoglossus kowalevskii*	Sko_Tdrd_XP_002737010	XP_002737010.1
Chordata	*Mus musculus*	Mmu_Tdrd_1	NP_001002238.1
	Mus musculus	Mmu_Tdrd_5	AAH99972.1
	Mus musculus	Mmu_Tdrd_6	AAI45249.1
	Mus musculus	Mmu_Tdrd_7	NP_666254.1
	Xenopus laevis	Xla_Tdrd_5	NP_001090599.1
	Xenopus laevis	Xla_Tdrd_7	NP_001084569.2
	Xenopus tropicalis	Xtr_Tdrd_6	XP_002938001.1
	Xenopus tropicalis	Xtr_Tdrd_7	NP_001011355.2
	Danio rerio	Dre_Tdrd_1	NP_001157500.1
	Danio rerio	Dre_Tdrd_5	AAI34986.1
	Danio rerio	Dre_Tdrd_6	NP_001182210.1
	Danio rerio	Dre_Tdrd_7	ABR24798.1
	Branchiostoma floridae	Bfl_Tdrd_XP_002608013	XP_002608013.1

	Branchiostoma floridae	Bfl_Tdrd_XP_002609625	XP_002609625.1
	Branchiostoma floridae	Bfl_Tdrd_7_XP_002590816	XP_002590816.1
Cnidaria	*Actinia tenebrosa*	Ate_Tdrd_XP_031549775	XP_031549775.1
	Hydra vulgaris	Hvu_Tdrd_1	XP_047129251.1
	Hydra vulgaris	Hvu_Tdrd_6	XP_047142151.1
	Hydra vulgaris	Hvu_Tdrd_7	XP_047145945.1
	Nematostella vectensis	Nve_Tdrd_XP_001627217	XP_001627217.1
	Nematostella vectensis	Nve_Tdrd_XP_001628171	XP_001628171.1
	Nematostella vectensis	Nve_Tdrd_EDO36108	EDO36108.1
Porifera	*Amphimedon queenslandica*	Aqu_Tdrd_1_XP_003388077	XP_003388077.1
	Amphimedon queenslandica	Aqu_Tdrd_XP_003384366	XP_003384366.1
	Oscarella lobularis1	Olo_Tdrd_1_AQX83031	AQX83031.1
Fungi	*(outgroup) Schizosaccharomyces pombe*	Spo_snd1	NP_588117.1

Table S8. GenBank accession numbers for sequences used for Boule amino acid alignments.

**Phylum**	**Species**	**Gene name in tree**	**GenBank #**
Annelida	*Enchytraeus coronatus*	Eco-boule1	OR750686
	Enchytraeus coronatus	Eco-boule2	OR750687
	Helobdella robusta	Hro_boule	XP_009028824.1
	Owenia fusiformis	Ofu_boule	CAH1789320.1
Platyhelminthes	*Echinococcus multilocularis*	Emu_boule	CDS41941.1
	Macrostomum lignano	Mli_boule1	ADL09421.1
	Macrostomum lignano	Mli_boule2	AEK69206.1
	Macrostomum lignano	Mli_boule3	AEK69207.1
	Schmidtea mediterranea	Sme_boule1	ANE21796.1
	Schmidtea mediterranea	Sme_boule2	ANE21797.1
Mollusca	*Aplysia californica*	Aca_boule	XP_005103136.2
	Crassostrea gigas	Cgi_boule	XP_034310513.1
	Crassostrea virginica	Cvi_boule	XP_022305710.1
	Octopus vulgaris	Ovu_boule	CAI9742253.1
	Patella vulgata	Pvu_boule	XP_050418578.1
	Pecten maximus	Pma_boule	XP_033761447.1
	Sepia pharaonis	Sph_boule	CAE1318733.1
Brachiopoda	*Lingula anatina*	Lan_boule	XP_013418885.1
Arthropoda	*Drosophila melanogaster*	Dme_boule	AAF50316.3
	Aedes albopictus	Aal_boule	XP_029725578.1
	Anopheles arabiensis	Aar_boule	XP_040155388.1
	Apis mellifera	Ame_boule	XP_003250213.1
	Culex quinquefasciatus	Cqu_boule	XP_038118889.1
	Nasonia vitripennis	Nvi_boule	XP_001599398.1
	Tribolium castaneum	Tca_boule	EFA05679.2

Echinodermata	*Strongylocentrotus purpuratus*	XP_030846218.1	Spu_boule
	Patiria miniata	Pmi_boule	XP_038044814.1
Hemichordata	*Saccoglossus kowalevskii*	Sko_boule	XP_006818817.1
Chordata	*Mus musculus*	Mmu_boule	NP_001354816.1
	Oryzias latipes	Ola_boule	ACU31026.1
Cnidaria	*Actinia tenebrosa*	Ate_boule	XP_031560060.1
	Nematostella vectensis	Nve_boule	XP_032243176.2
Fungi	*(outgroup) Saccharomyces cerevisiae*	Sce_HRP1	CAA99142.1

Table S9. GenBank accession numbers for sequences used for RRM domain protein Bruno amino acid alignments.

**Phylum**	**Species**	**Gene name in tree**	**GenBank #**
Annelida	*Capitella teleta*	Cte_ELU11974	ELU11974.1
	Capitella teleta	Cte_ELT93212	ELT93212.1
	Enchytraeus coronatus	Eco_bruno1	OR750688
	Enchytraeus coronatus	Eco_bruno2	OR750689
	Enchytraeus coronatus	Eco_bruno3	OR750690
	Enchytraeus coronatus	Eco_bruno4	OR750691
	Helobdella robusta	Hro_XP_009029960	XP_009029960.1
	Helobdella robusta	Hro_XP_009009284	XP_009009284.1
	Helobdella robusta	Hro_ESN89902	ESN89902.1
	Helobdella robusta	Hro_XP_009031985	XP_009031985.1
	Malacoceros fuliginosus	Mfu_CUGBP_elav_like_3	QNN94692.1
	Owenia fusiformis	Ofu_celf	QWY04388.1
	Platynereis dumerilii	Pdu_bruno	CCK33023.1
Mollusca	*Crassostrea gigas*	Cgi_CUGBP_elav_like_2	XP_034333010.1
	Crassostrea gigas	Cgi_CUGBP_elav_like_3B	XP_034317472.1
	Lamellibrachia satsuma	Lsa_CUGBP_elav_like_4	KAI0219664.1
	Lottia gigantea	Lgi_XP_009047452	XP_009047452.1
	Lottia gigantea	Lgi_XP_009055917	XP_009055917.1
	Patella vulgata	Pvu_CUGBP_elav_like_2	XP_050392146.2
	Patella vulgata	Pvu_CUGBP_elav_like_3B	XP_050395857.1
	Pecten maximus	Pma_CUGBP_elav_like_2	XP_033740337.1
	Pecten maximus	Pma_CUGBP_elav_like_4	XP_033725903.1
Brachiopoda	*Lingula anatina*	Lan_CUGBP_elav_like_2	XP_013383614.1
	Lingula anatina	Lan_CUGBP_elav_like_3A	XP_013408611.1
Arthropoda	*Drosophila melanogaster*	Dme_bruno1_isoformB	NP_723737.1
	Drosophila melanogaster	Dme_bruno2_isoformE	NP_001036356.1
	Drosophila melanogaster	Dme_bruno3_isoformA	NP_729923.1
	Tribolium castaneum	Tca_CUGBP_elav_like_2	XP_015838769.1
	Tribolium castaneum	Tca_CUGBP_elav_like_4	XP_015839523.1
	Ixodes scapularis	Isc_XP_002436197	XP_002436197.1

	Ixodes scapularis	Isc_XP_002404307	XP_002404307.1
	Limulus polyphemus	Lpo_CUGBP_elav_like_2	XP_022236600.1
	Limulus polyphemus	Lpo_CUGBP_elav_like_4	XP_013783377.2
Echinodermata	*Lytechinus variegatus*	Lva_CUGBP_elav_like_3B	XP_041459854.1
	Strongylocentrotus purpuratus	Spu_CUGBP_elav_like_1A	XP_782270.3
	Strongylocentrotus purpuratus	Spu_CUGBP_elav_like_2	XP_030844245.1
	Strongylocentrotus purpuratus	Spu_CUGBP_elav_like_3	XP_030829134.1
	Strongylocentrotus purpuratus	Spu_CUGBP_elav_like_4	XP_781047.2
Hemichordata	*Saccoglossus kowalevskii*	Sko_bruno_like	NP_001161510.1
	Saccoglossus kowalevskii	Sko_bruno2_like	XP_002736280.1
Chordata	*(outgroup) Mus musculus*	Mmu_elav_1	NP_034615.2
	Mus musculus	Mmu_CUGBP_elav_like_1	NP_059064.2
	Mus musculus	Mmu_CUGBP_elav_like_2	NP_001103698.1
	Mus musculus	Mmu_CUGBP_elav_like_3	NP_766022.1
	Mus musculus	Mmu_CUGBP_elav_like_4	XP_036016831.1
	Mus musculus	Mmu_CUGBP_elav_like_5	NP_795928.2
	Mus musculus	Mmu_CUGBP_elav_like_6	NP_780444.2
	Xenopus tropicalis	Xtr_CUGBP_elav_like_3	NP_001016491.1
	Xenopus tropicalis	Xtr_CUGBP_elav_like_4	NP_001072378.1
	Xenopus tropicalis	Xtr_CUGBP_elav_like_5	NP_001090639.1
	Danio rerio	Dre_CUGBP_elav_like_1	NP_571688.1
	Danio rerio	Dre_CUGBP_elav_like_2	NP_919382.1
	Danio rerio	Dre_CUGBP_elav_like_3	NP_571569.2
	Danio rerio	Dre_CUGBP_elav_like_4	NP_001002562.1
	Danio rerio	Dre_CUGBP_elav_like_5A	NP_001124260.1
	Danio rerio	Dre_celf3	XP_001335316.2
	Danio rerio	Dre_CUGBP_elav_like_6	XP_001334980.2
	Branchiostoma floridae	Bfl_CUGBP_elav_like_2	XP_035664982.1
	Branchiostoma floridae	Bfl_CUGBP_elav_like_4	XP_035668685.1
	Branchiostoma floridae	Bfl_CUGBP_elav_like_3A	XP_035668681.1
	Branchiostoma floridae	Bfl_XP_002588859	XP_002588859.1
	Branchiostoma floridae	Bfl_XP_002588860	XP_002588860.1
Cnidaria	*Nematostella vectensis*	Nve_CUGBP_elav_like_2	XP_048578496.1
	Nematostella vectensis, ,	Nve_XP_001627659	XP_001627659.1
	Nematostella vectensis	Nve_XP_001622676	XP_001622676.1
	Nematostella vectensis	Nve_XP_001621619	XP_001621619.1
Protista	*Monosiga brevicollis*	Mbr_XP_001745306	XP_001745306.1

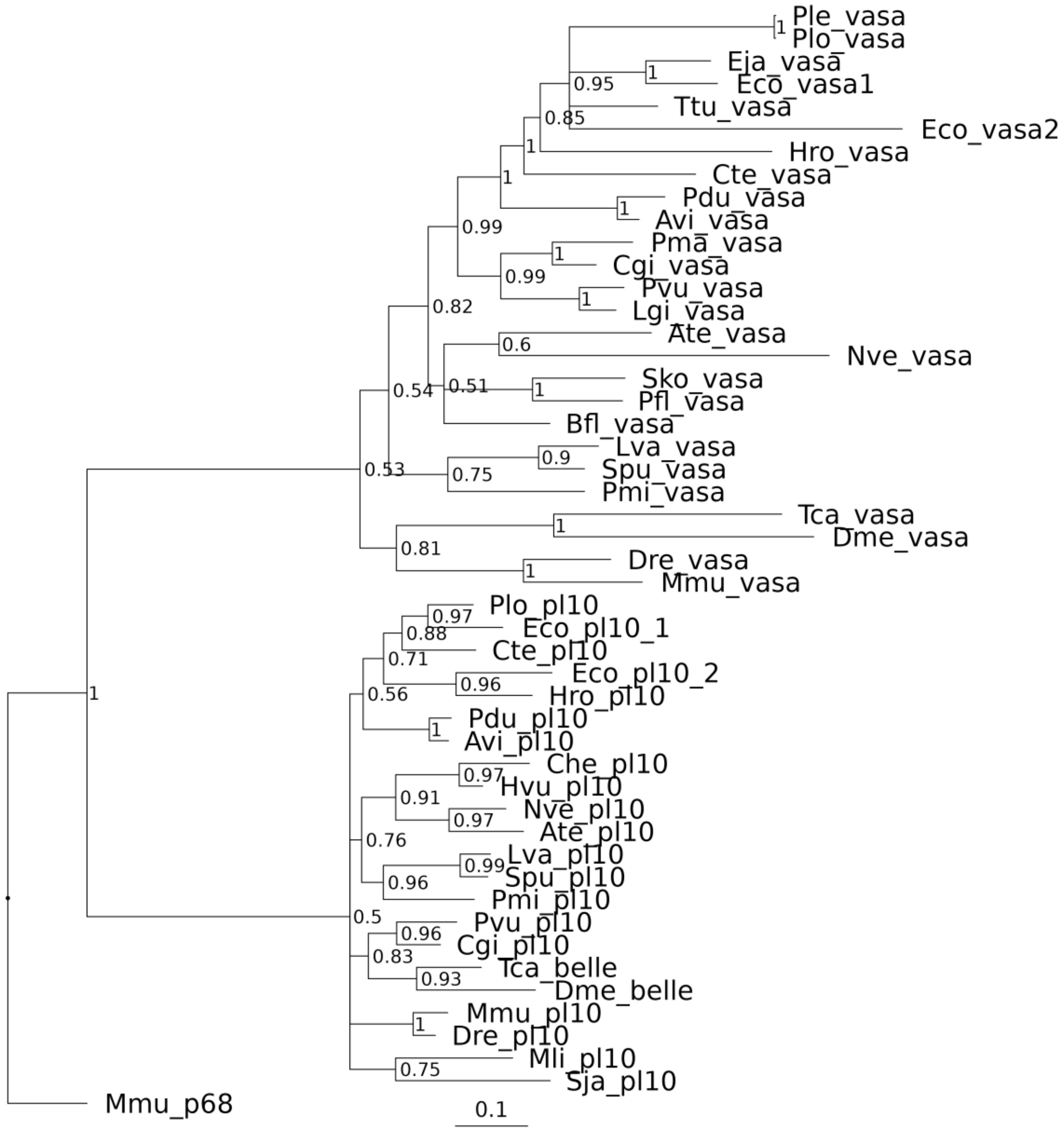

Figure S1. Phylogenetic analysis of *Enchytraeus coronatus Vasa* and *Pl10* homologs. Bayesian consensus tree of the Helicase domains of metazoan *Vasa* and *Pl10* genes.

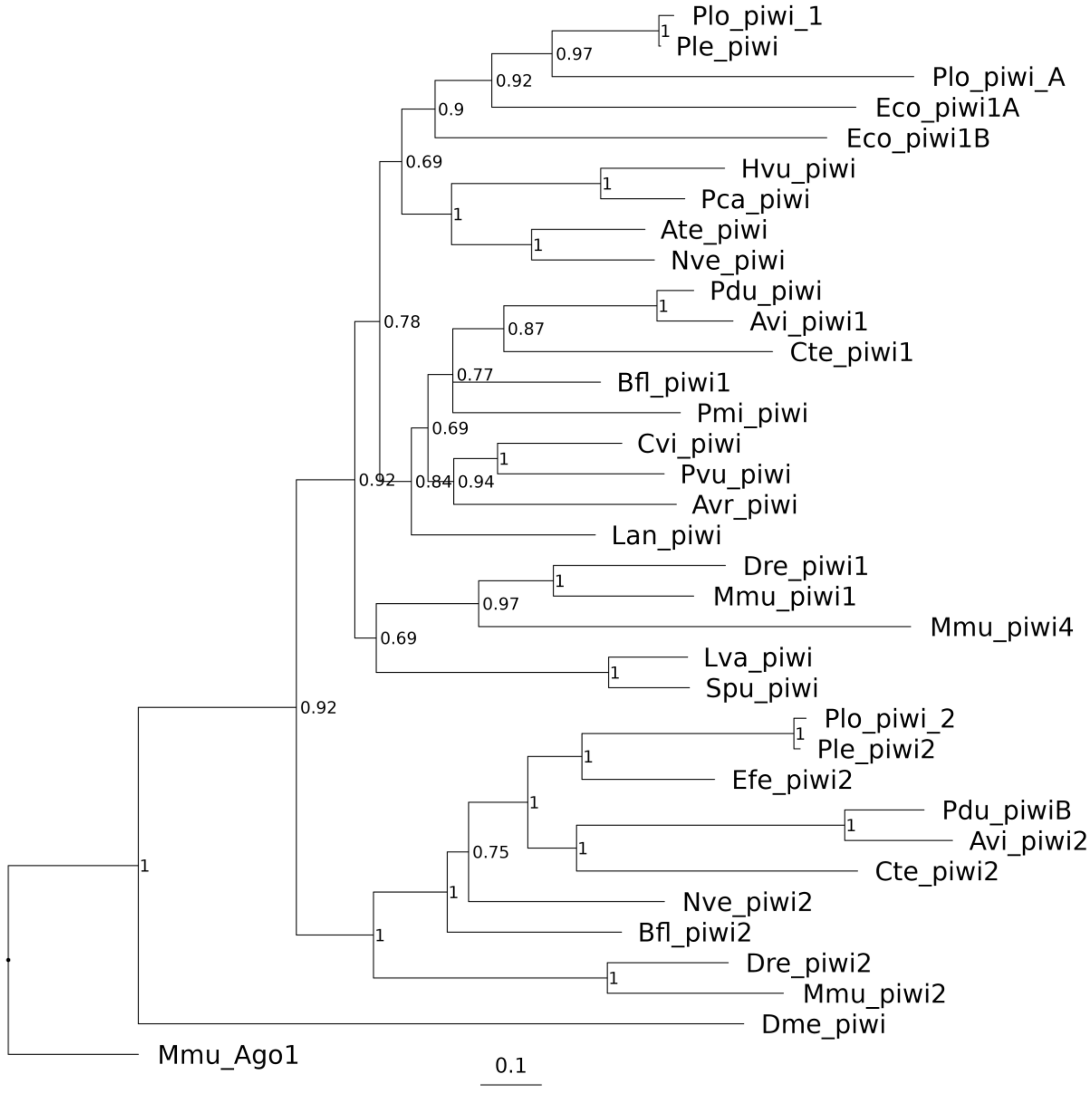

Figure S2. Phylogenetic analysis of *Enchytraeus coronatus Piwi* homologs. Bayesian consensus tree of the Piwi domain of metazoan *Piwi* genes.

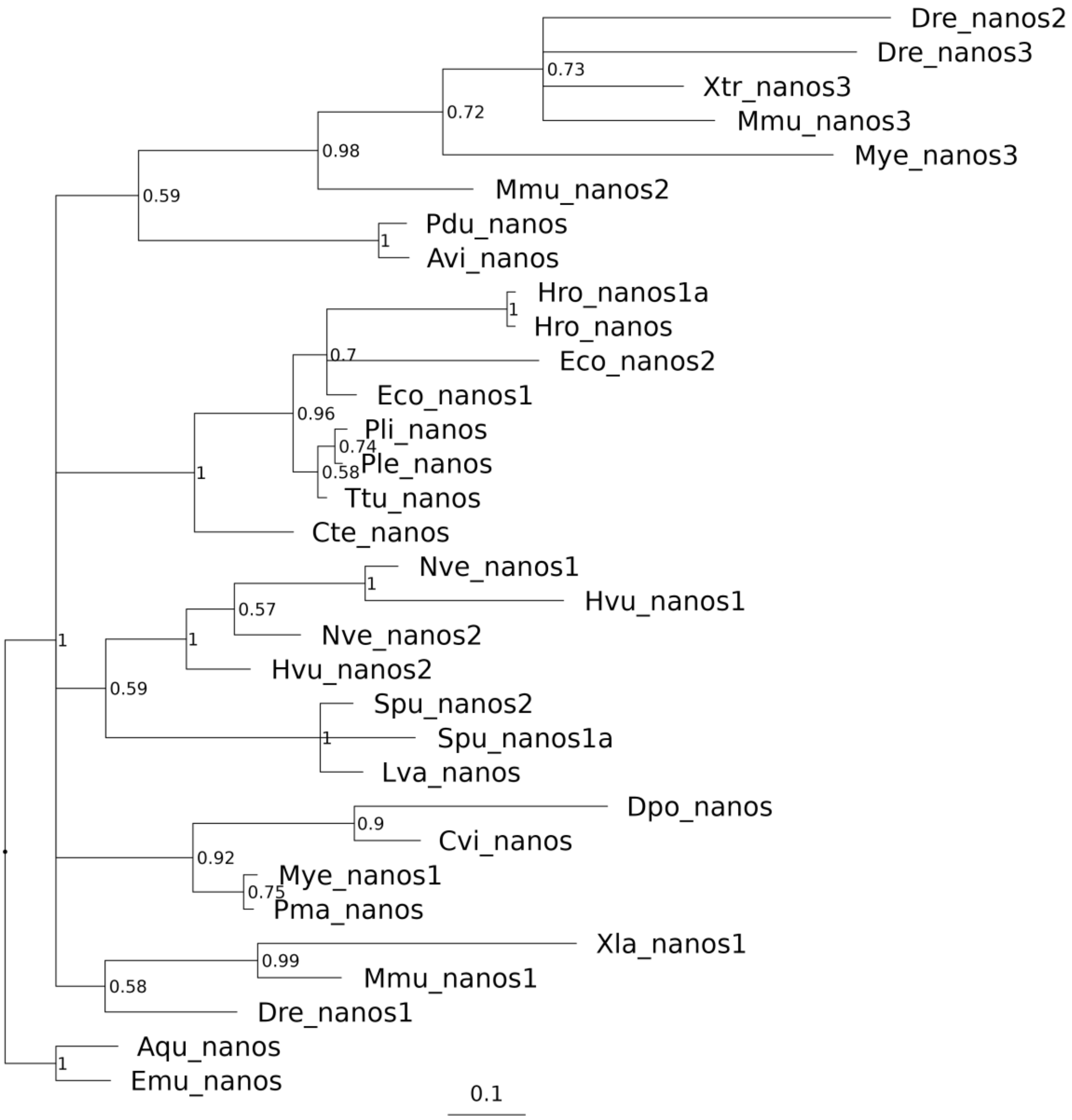

Figure S3. Phylogenetic analysis of *Enchytraeus coronatus Nanos* homologs. Bayesian consensus tree of the Nanos (CCHC)-type zinc-finger domain of metazoan *Nanos* genes.

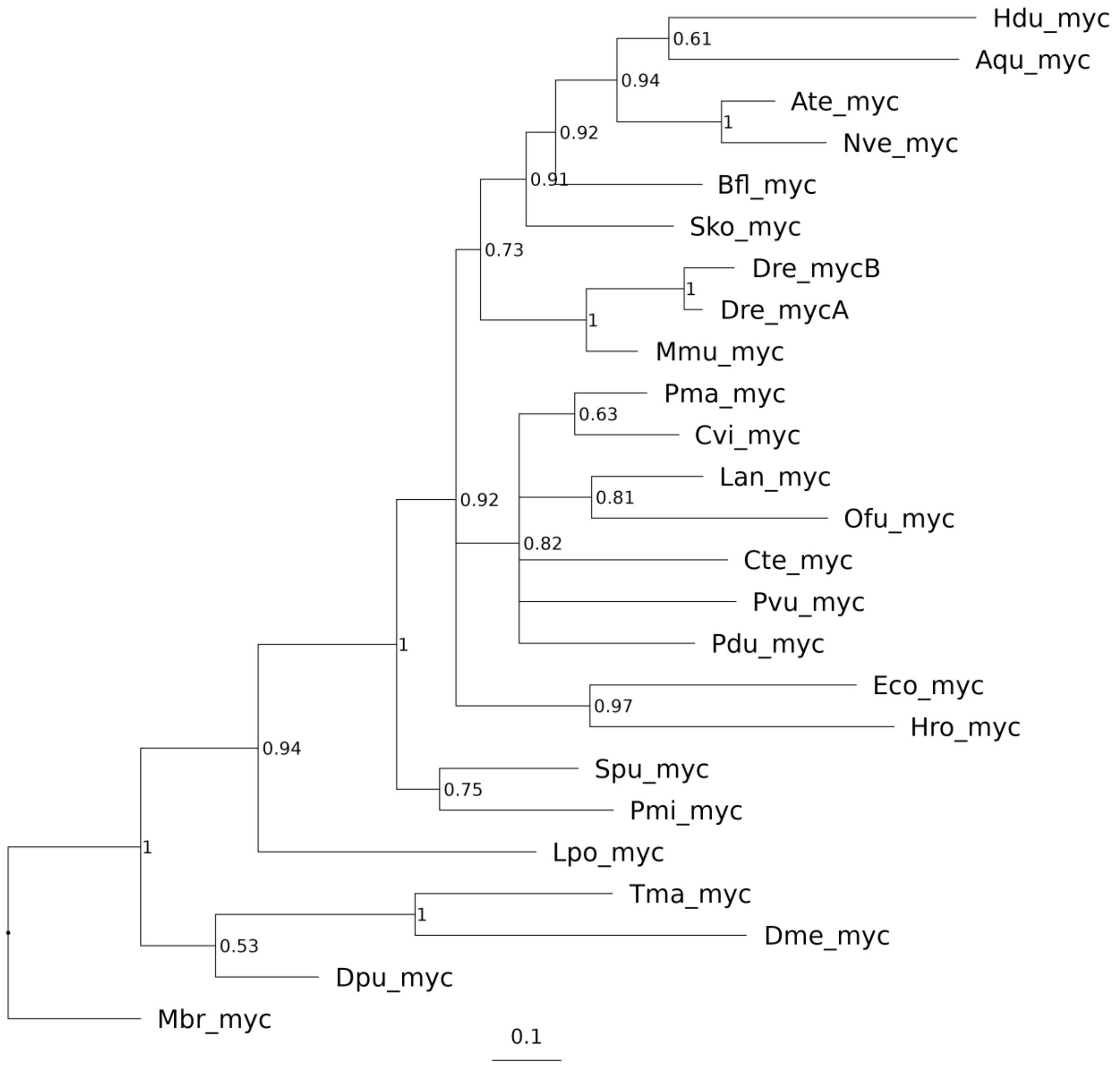

Figure S4. Phylogenetic analysis of *Enchytraeus coronatus Myc* homolog. Bayesian consensus tree of the Myc-type basic helix-loop-helix (bHLH) domain of metazoan *Myc* genes.

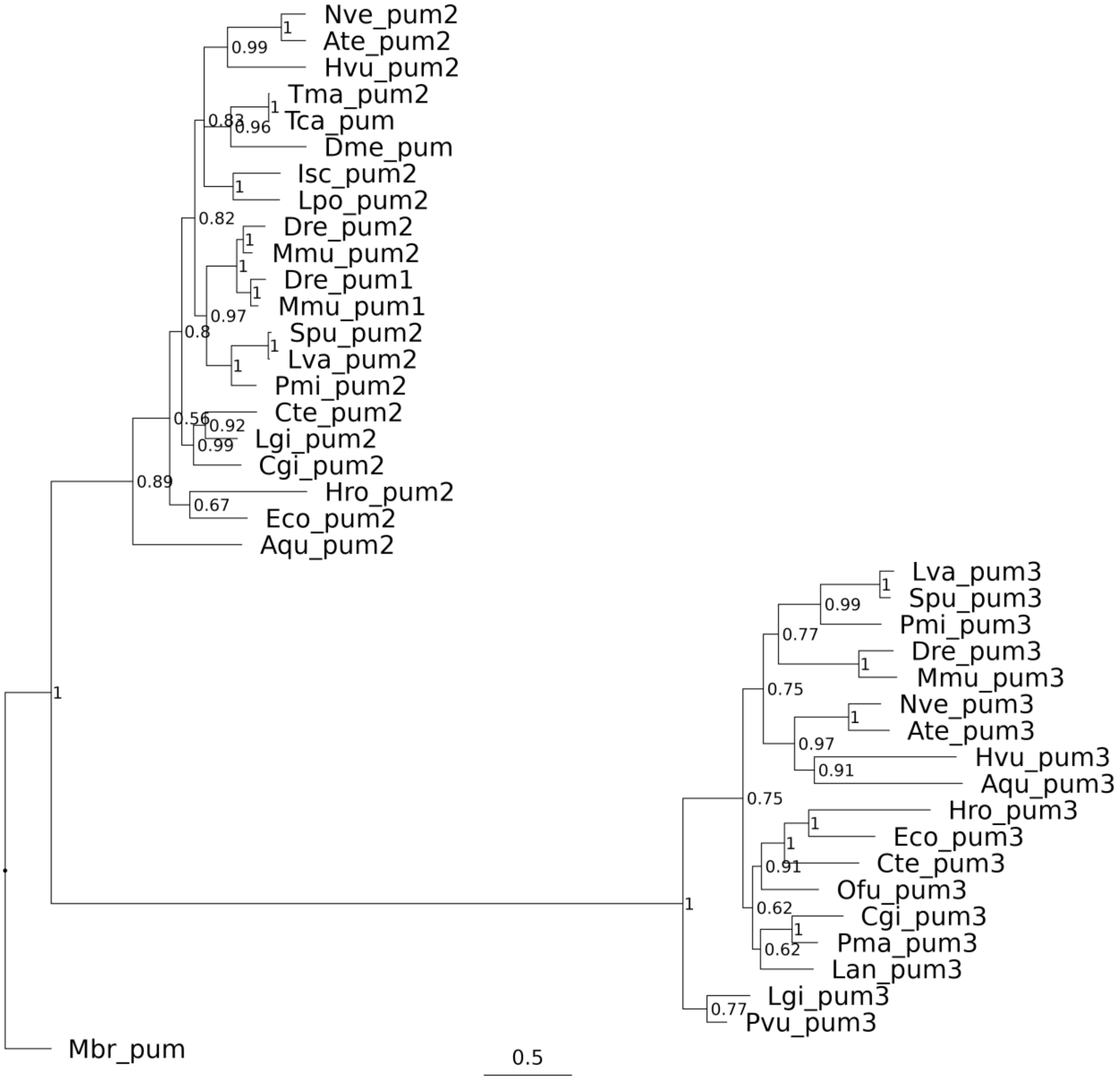

Figure S5. Phylogenetic analysis of *Enchytraeus coronatus Pumilio* homologs. Bayesian consensus tree of the Pumilio domain of metazoan *Pumilio* genes.

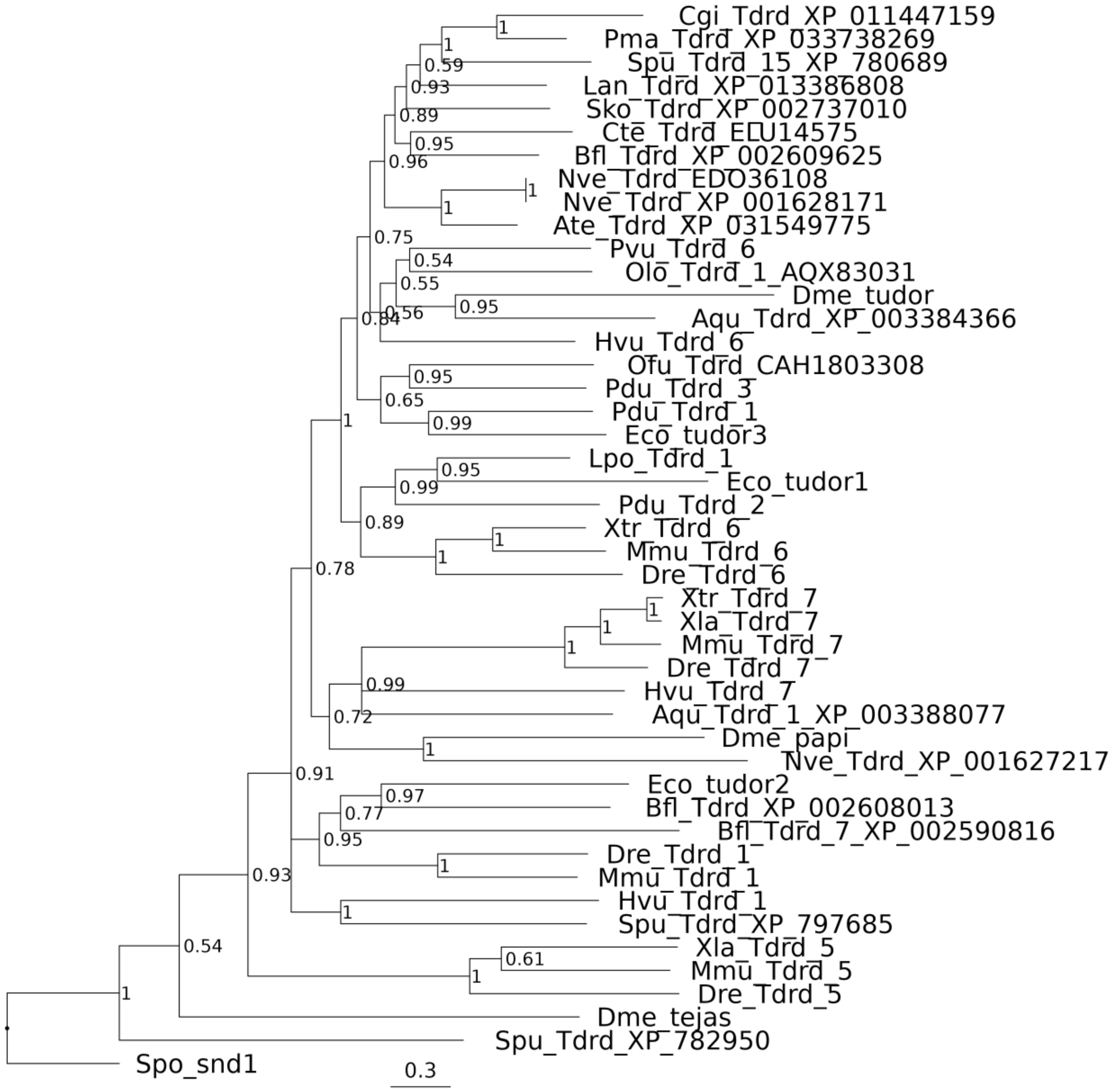

Figure S6. Phylogenetic analysis of *Enchytraeus coronatus Tudor* homologs. Bayesian consensus tree of the full-length alignment of metazoan *Tudor* genes.

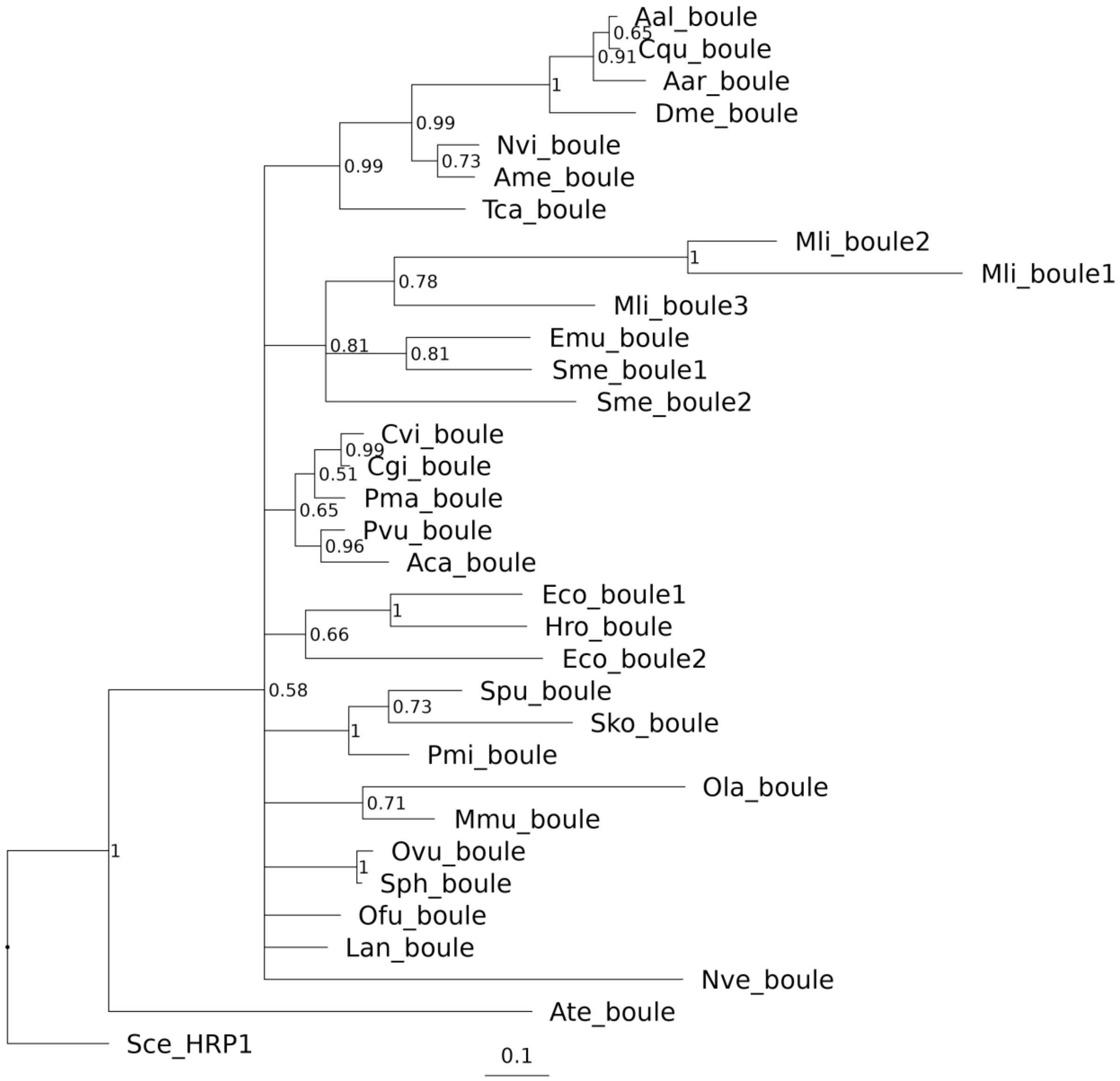

Figure S7. Phylogenetic analysis of *Enchytraeus coronatus Boule* homologs. Bayesian consensus tree of the Boule RRM domain of metazoan *Boule* genes.

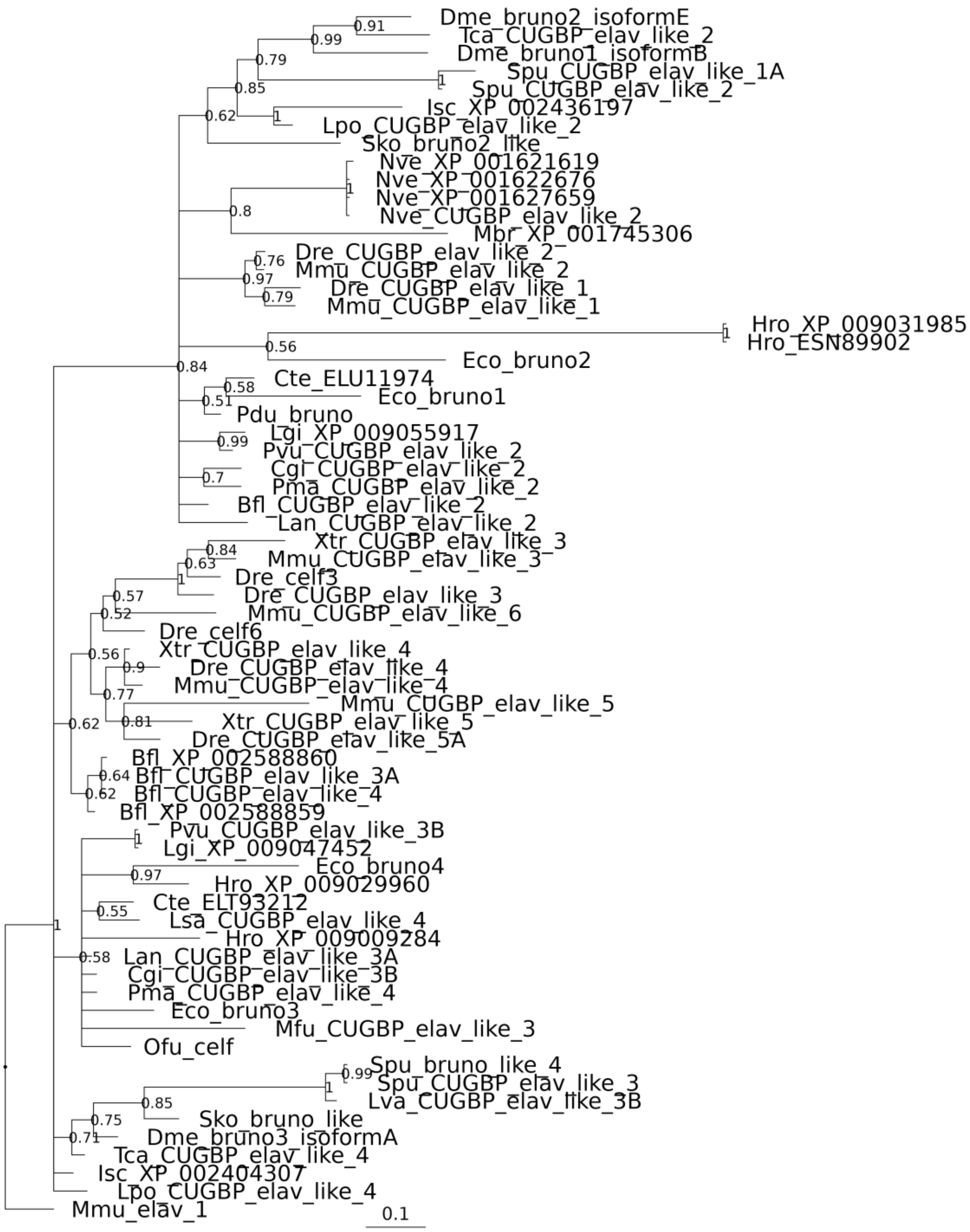

Figure S8. Phylogenetic analysis of *Enchytraeus coronatus Bruno* homologs. Bayesian consensus tree of the Bruno RRM domain of metazoan *Bruno/CUGBP-Elav-like* genes.

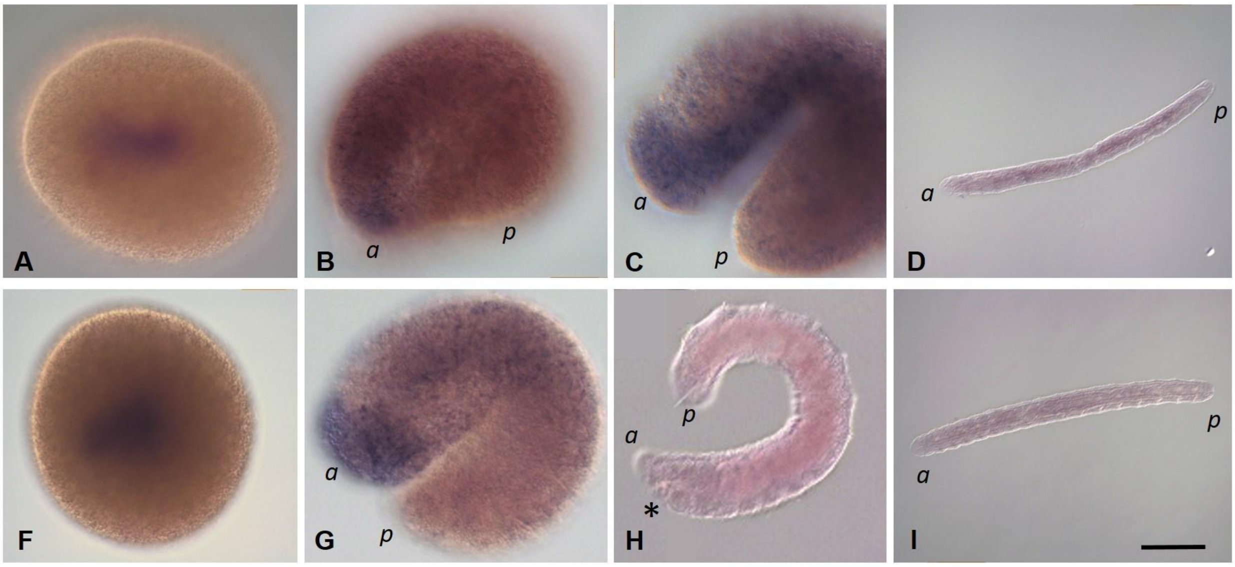

Figure S9. *Eco-boule1* and *Eco-boule2* expression patterns. *Eco-boule1* (**A**–**D**) and *Eco-boule2* (**F**–**I**) expression patterns. (**A**,**F**) mRNA of both *Boule* orthologs is detected in oocytes. (**B**) *Eco-boule1* expression in the descendants of the animal micromeres and germband cells. (**C**) *Eco-boule1* expression becomes particularly prominent in cells of the animal pole and the ventral side of the anterior part of the embryo. (**G**) *Eco-boule2* expression in superficial cells is very weak, except for a domain in the most anterior part of the embryo. (**D**,**I**) In juvenile, no expression of identified *Boule* homologs is detected by WMISH. Asterisk marks the mouth position; *a* and *p* mark the anterior and posterior end, respectively. Scale bar, 50 µm for all panels except **D**, **H** and **I**. Scale bar in **D** and **I**, 170 µm; in **H**, 80 µm.
